# Co-depletion of NIPBL and WAPL balance cohesin activity to correct gene misexpression

**DOI:** 10.1371/journal.pgen.1010528

**Published:** 2022-11-30

**Authors:** Jennifer M. Luppino, Andrew Field, Son C. Nguyen, Daniel S. Park, Parisha P. Shah, Richard J. Abdill, Yemin Lan, Rebecca Yunker, Rajan Jain, Karen Adelman, Eric F. Joyce

**Affiliations:** 1 Department of Genetics, Perelman School of Medicine, University of Pennsylvania, Philadelphia, Pennsylvania, United States of America; 2 Penn Epigenetics Institute, University of Pennsylvania, Philadelphia, Pennsylvania, United States of America; 3 Department of Biological Chemistry and Molecular Pharmacology, Blavatnik Institute, Harvard Medical School, Boston, Massachusetts, United States of America; 4 Ludwig Center at Harvard, Boston, Massachusetts, United States of America; 5 Department of Cell and Developmental Biology, Department of Medicine, Institute of Regenerative Medicine, Penn Cardiovascular Institute, Perelman School of Medicine, University of Pennsylvania, Philadelphia, Pennsylvania, United States of America; 6 The Eli and Edythe L. Broad Institute, Cambridge, Massachusetts, United States of America; Geisel School of Medicine at Dartmouth, UNITED STATES

## Abstract

The relationship between cohesin-mediated chromatin looping and gene expression remains unclear. NIPBL and WAPL are two opposing regulators of cohesin activity; depletion of either is associated with changes in both chromatin folding and transcription across a wide range of cell types. However, a direct comparison of their individual and combined effects on gene expression in the same cell type is lacking. We find that NIPBL or WAPL depletion in human HCT116 cells each alter the expression of ~2,000 genes, with only ~30% of the genes shared between the conditions. We find that clusters of differentially expressed genes within the same topologically associated domain (TAD) show coordinated misexpression, suggesting some genomic domains are especially sensitive to both more or less cohesin. Finally, co-depletion of NIPBL and WAPL restores the majority of gene misexpression as compared to either knockdown alone. A similar set of NIPBL-sensitive genes are rescued following CTCF co-depletion. Together, this indicates that altered transcription due to reduced cohesin activity can be functionally offset by removal of either its negative regulator (WAPL) or the physical barriers (CTCF) that restrict loop-extrusion events.

## Author summary

The cohesin protein complex plays a key role in the formation and dynamics of DNA loops in the cell nucleus. How these loops guide genes to distal enhancers and influence their expression remains unclear. We depleted two opposing regulators of cohesin activity, NIPBL and WAPL, to compare their independent and shared effects on nascent gene expression in human cells. We show patterns of dysregulation that are both unique and highly similar between the depletions, with some large genomic regions showing amplified sensitivity to both. Finally, we show that co-depletion of NIPBL and WAPL restores the majority of gene misexpression as compared to either knockdown alone. This indicates that the balance between the factors, rather than their absolute levels, is especially important for regulating cohesin dynamics and DNA looping that can influence gene expression.

## Introduction

The dynamics underlying cohesin-mediated chromatin looping depend on the interplay between two mutually exclusive regulators of cohesin, Nipped-B-like protein (NIPBL) and Wings apart-like protein homolog (WAPL) [1–3]. NIPBL, together with MAU2, have been proposed to both load cohesin and activate its ATPase domain to initiate loop extrusion [4,5,3,6,7]. In contrast, WAPL removes cohesin from chromatin, limiting its residence time to minutes, thus restricting the size of loops across the genome [8–10,2,11,12]. While loss of cohesin or its regulators have been shown to each lead to widespread, albeit modest, effects on gene expression across a wide range of systems and cell types [12–18], the comparative effects of NIPBL and WAPL perturbation on gene regulation in the same cell type has yet to be studied. Furthermore, while co-depletion of MAU2 and WAPL have been shown to rescue chromatin misfolding at the population level [2], it remains unclear whether restoring chromatin looping is sufficient for normal gene regulation.

In this study, we address these questions by depleting NIPBL and/or WAPL from chromatin in human cells and compare their effects on nascent gene expression. Importantly, we deplete NIPBL or WAPL to levels that alter chromatin folding at the TAD and sub-TAD scale by Oligopaint fluorescence *in situ* hybridization (FISH) without affecting mitosis or cell growth. We find that NIPBL or WAPL depletion is associated with misexpression of many genes unique to either condition as well as some shared differentially expressed genes between them. Interestingly, the differentially expressed genes shared between the two perturbations are mostly altered in the same direction, suggesting these genes are equally sensitive to both increased and decreased cohesin activity. Nonetheless, NIPBL- and WAPL-specific sensitive genes have many shared features, including proximity to loop anchors, cohesin binding sites, and each other. Indeed, differentially expressed genes are clustered within TADs and exhibit coordinated misexpression, suggesting there are differential genomic regions with increased sensitivity to altered levels of cohesin.

Remarkably, co-depletion of both cohesin regulators rescued gene misexpression compared to either single knockdown. A similar rescue of gene expression was observed in NIPBL and CTCF co-depleted cells. We propose that a balance, rather than absolute levels, of cohesin loading, unloading, and processivity may be essential for normal cohesin function. Together, these studies provide insights into how cohesin is dynamically regulated by opposing cofactors to organize chromatin and facilitate proper gene regulation.

## Results

### Partial depletion of NIPBL or WAPL separate the role of cohesin in chromatin folding from chromosome segregation

**Fig 1:**
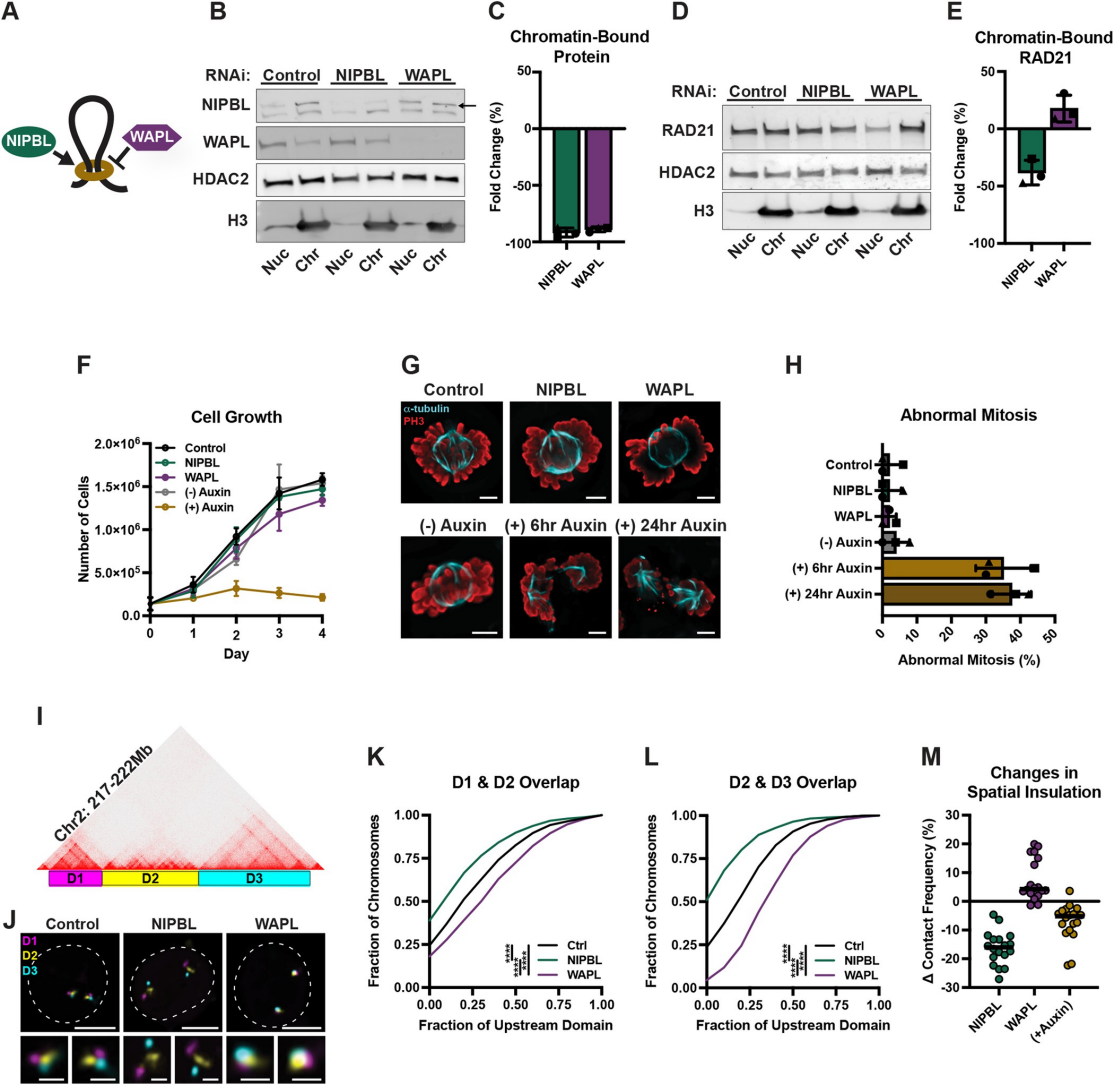
Partial depletion of NIPBL or WAPL separate the role of cohesin in chromatin folding from chromosome segregation. (A) Cartoon depicting the roles of the two opposing cohesin regulators; NIPBL loads cohesin onto chromatin and is required for loop extrusion whereas WAPL opens the ring and removes it. (B) Fluorescent western blot to NIPBL (top band, see arrow) and WAPL in nuclear (nuc) and chromatin-bound (chr) subcellular protein fractionations of RNAi control, NIPBL, or WAPL depleted HCT116 cells. All bands are from the same blot. (C) Mean fold change (%) of NIPBL and WAPL bound to chromatin in each respective knockdown. Each symbol represents a biological replicate, error bars represent standard deviation. (D) Fluorescent western blot to RAD21 in nuclear (nuc) and chromatin-bound (chr) subcellular protein fractionations of RNAi control, NIPBL, or WAPL depleted HCT116 cells. All bands are from the same blot. (E) Mean fold change (%) of RAD21 bound to chromatin in each respective knockdown. Each symbol represents a biological replicate, error bars represent standard deviation. (F) Cell growth measured in 24-hour increments following RNAi or auxin treatment. Each bar represents the mean of 3 biological replicates and error bars represent the standard deviation. (G) Representative immunofluorescence images of mitotic cells stained for α-tubulin (cyan) and phospho-Histone H3 (PH3; red). Top row are HCT116 cells following 72 hour treatment with RNAi against control, NIPBL, or WAPL. Bottom row are HCT116-RAD21-AID cells -/+ auxin for 6 or 24 hours. Scale bar, 5μm. (H) Average percentage of abnormal mitotic cells in RNAi control, NIPBL, or WAPL depleted HCT116 cells and HCT116-RAD21-AID cells -/+ auxin for 6 or 24 hours. Each symbol represents a biological replicate, error bars represent standard deviation. (I) Oligopaint design for three neighboring domains at chr2:217-222Mb. (J) Representative FISH images for three domains at chr2:217-222Mb in RNAi control, NIPBL, and WAPL depleted HCT116 cells. Dashed line represents nuclear edge, scale bar, 5μm (above) or 1μm (below). (K) Cumulative frequency distribution of overlap between the neighboring domains D1 and D2 on chr2 in RNAi control (n = 1,170 chromosomes), NIPBL (n = 1,177 chromosomes), or WAPL (n = 1,136 chromosomes) depleted HCT116 cells. Two-tailed Mann-Whitney test, **** p < 0.0001. (L) Cumulative frequency distribution of overlap between the neighboring domains D2 and D3 on chr2 in RNAi control (n = 1,202 chromosomes), NIPBL (n = 1,284 chromosomes), or WAPL (n = 1,149 chromosomes) depleted HCT116 cells. Two-tailed Mann-Whitney test, **** p < 0.0001. (M) Change in contact frequency across 18 domain pairs in NIPBL, or WAPL depleted HCT116 cells and HCT116-RAD21-AID cells treated with auxin for 6 hours. Each dot represents the median of ≥ 4 biological replicates at each locus.

We depleted NIPBL or WAPL for 72 hours using a pool of small interfering RNAs (siRNAs) in human HCT116 cells (Figs 1A, S1A and S1B). Quantitative PCR indicated that we achieved 56–79% RNA depletion of each factor (S1B Fig). To more accurately measure the amount of NIPBL and WAPL on chromatin at the 72-hour timepoint in which we subsequently measured transcription and chromatin folding, we performed subcellular protein fractionation followed by quantitative western blotting and measured a robust 92% and 89% reduction in chromatin-bound levels, respectively (Fig 1B and 1C).

Consistent with their known roles in cohesin loading and unloading, siNIPBL resulted in a 38% depletion of chromatin-bound RAD21 levels whereas siWAPL increased chromatin-bound RAD21 levels by 18% (Fig 1D and 1E). Importantly, NIPBL or WAPL depletion did not change the growth rate of cells over the course of four cell divisions and did not alter mitotic progression, chromosome segregation, or the frequency of mitotic entry under these conditions (Figs 1F–1H, S1C and S1D). As a control, near-complete loss of RAD21 (<10% remaining on chromatin; S1E and S1F Fig) via auxin-inducible degradation (AID) prompted growth arrest after the first cell division (Fig 1F) and resulted in a higher mitotic index with increased morphological abnormalities consistent with chromosome segregation errors (Figs 1G–1H, S1C and S1D). These results confirm that cohesin is essential for normal mitotic progression in HCT116 cells and further suggest that cells do not require the full complement of NIPBL or WAPL for cell growth or fidelity.

Next, to confirm that our depletion levels of NIPBL or WAPL are sufficient to alter chromatin folding, we used an Oligopaint fluorescence *in situ* hybridization (FISH)-based assay that we previously developed to quantify the frequency of interactions across domain boundaries as measured by the extent of spatial overlap between adjacent probes [19]. We first labeled three consecutive domains on chromosome 2 that had strong intervening boundaries (20th and 6th percentiles, as measured by Hi-C insulation scores) (Fig 1I). Neighboring domains exhibited less spatial overlap in cells depleted of NIPBL than in control cells, consistent with chromatin misfolding of the labelled locus (Fig 1J–1L). The extent of spatial separation was similar to that observed after acute depletion (6 hour) of RAD21 (S1G–S1I Fig). In contrast, WAPL depletion led to increased interactions across both domain boundaries (Fig 1J–1L).

To compare partial depletion of NIPBL and WAPL to near complete loss of RAD21, we expanded our FISH assay to label sixteen additional domain or sub-domain boundaries (S1J Fig). The Oligopaint probes spanned regions of different boundary strengths (defined by their insulation score), gene densities, and chromatin types. We used a recently developed high-throughput FISH platform, called HiDRO, to image at least four biological replicates of each FISH reaction in parallel [20]. We defined a contact cutoff of 250 nm based on the resolution of our microscope to quantify interactions across domain boundaries. We found that cohesin loss by either siNIPBL or RAD21 AID decreased the contact frequencies across all boundaries assayed with similar yet variable locus sensitivities (Figs 1M and S1K–S1M). Specifically, we observed a 5–28% and 2–22% decrease in contact frequency after NIPBL depletion and RAD21 AID, respectively (Figs 1M and S1M). WAPL depletion increased contact across most boundaries, with a 1–20% increase in contact frequency in 16/18 domain pairs (Figs 1M and S1M). Therefore, our robust depletion of either protein was sufficient to alter chromatin folding by FISH without affecting cell growth or proliferation.

### NIPBL and WAPL regulate the expression of unique and shared sets of genes

Our partial depletion system allowed us to determine the extent of gene expression changes with robust reductions of NIPBL or WAPL from chromatin independent from chromosome segregation errors. We performed precision nuclear run-on sequencing (PRO-seq) to map the locations of active RNA polymerases and to determine levels of nascent transcription across two biological replicates. Given the reproducibility between our replicates (Spearman’s rho ≥0.95), we merged the data within each condition for downstream analyses.

**Fig 2:**
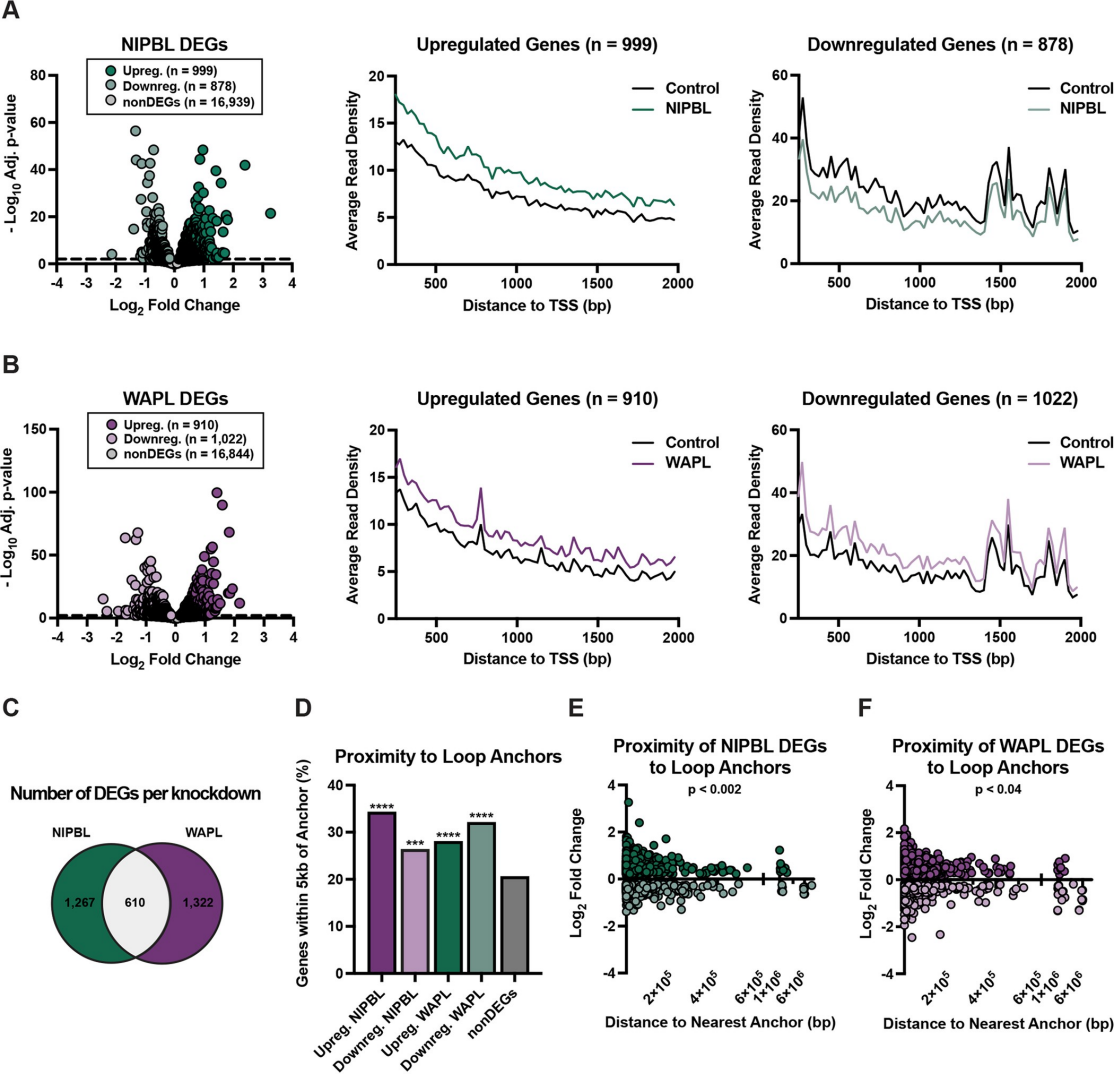
NIPBL and WAPL regulate the expression of unique and shared sets of genes. (A) The log_2_(fold change) of genes after NIPBL knockdown versus their significance. DEGs are in green (999 up, 878 down) and non-significantly changed genes (nonDEGs, adjusted p-value > 0.01) are in grey. Average read density over genes from transcriptional start site (TSS) upregulated (middle) or downregulated (right) after NIPBL knockdown. (B) The log_2_(fold change) of genes after WAPL knockdown versus their significance. DEGs are in purple (910 up, 1022 down) and nonDEGs (adjusted p-value > 0.01) are in grey. Average read density over genes from TSS upregulated (middle) or downregulated (right) after WAPL knockdown. (C) Venn diagram of the number of NIPBL, WAPL, and shared DEGs. (D) Percentage of up, down, NIPBL, WAPL, or nonDEGs with a TSS within 5kb of a loop anchor. Fisher’s exact test compared to nonDEGs, **** p < 0.0001, *** p = 0.0002. (E) Distance from each NIPBL DEG TSS to the nearest loop anchor versus the fold change of the gene. Spearman correlation, p = 0.0018. (F) Distance from each WAPL DEG TSS to the nearest loop anchor versus the fold change of the gene. Spearman correlation, p = 0.037.

To define differentially expressed genes (DEGs), we applied the DESeq2 algorithm and further filtered significant DEGs for a minimum adjusted p-value of 0.01. We identified 1,877 and 1,932 DEGs after NIPBL or WAPL depletion, respectively (Fig 2A and 2B). Similar to previous reports in other cell types [12–18], most changes were modest, and >95% of the DEGs had less than a two-fold change in expression (Fig 2A and 2B**).** Genes were approximately equally up- and downregulated in each knockdown condition (53% upregulated and 47% downregulated DEGs after NIPBL knockdown; 47% upregulated and 53% downregulated DEGs after WAPL knockdown).

We compared our list of NIPBL- and WAPL-sensitive genes to the 4,195 DEGs following acute RAD21 depletion in the same cell line [15]. Despite differences in the gene target and timing (72 versus 6 hours), a significantly greater than expected number of DEGs were shared after acute RAD21 degradation to that of NIPBL depletion (n = 578 genes, P<0.0001), further validating our DEG designations. Indeed, DEGs from both datasets were enriched in the same top four Gene Ontology (GO) terms for biological processes (S2A and S2B Fig and S1 and S2 Tables). In contrast to NIPBL, however, genes sensitive to WAPL depletion did not significantly overlap with those misexpressed following RAD21 degradation (n = 405 genes, P = 0.51). Consistent with this finding, >70% of the DEGs were unique to either NIPBL or WAPL depletion with the top GO terms differing between the conditions, indicating that not only different genes but also different pathways were predominantly affected by the two knockdowns (S2A and S2C Fig and S1 and S3 Tables). This suggests that many genes are uniquely sensitive to either loss (NIPBL and RAD21) or gain (WAPL) of cohesin activity. However, we also identified 610 genes that were indeed sensitive to either NIPBL or WAPL depletion. Interestingly, >80% of these genes (473 genes) were changed in the same direction, with equal rates of up- and downregulation observed (S2D Fig). Taken together, these data show that while many genes are uniquely sensitive to NIPBL or WAPL depletion, those that are shared between conditions tend to change in the same direction (S2D Fig).

While it remains unclear if DEGs are the result of direct or indirect misregulation, the promoters of DEGs were significantly enriched for both RAD21 and CTCF as compared with the promoters of nonDEGs (S2E Fig). We also found that 95% of the DEGs in either condition were within 200 kb of a loop anchor identified by *in situ* Hi-C data from parental HCT116 cells. This included ~30% of genes that were within 5 kb of a loop anchor. In comparison, only 20% of the nonDEGs were found within 5kb of a loop anchor (Fig 2D). Moreover, we found that genes closer to anchors tended to have a greater fold change in expression (Fig 2E and 2F). These results mimic our previous analyses following acute loss of RAD21 [19], highlighting a general signature of cohesin dysfunction across multiple components.

### Cohesin-sensitive genes are clustered and coordinated within TADs

**Fig 3:**
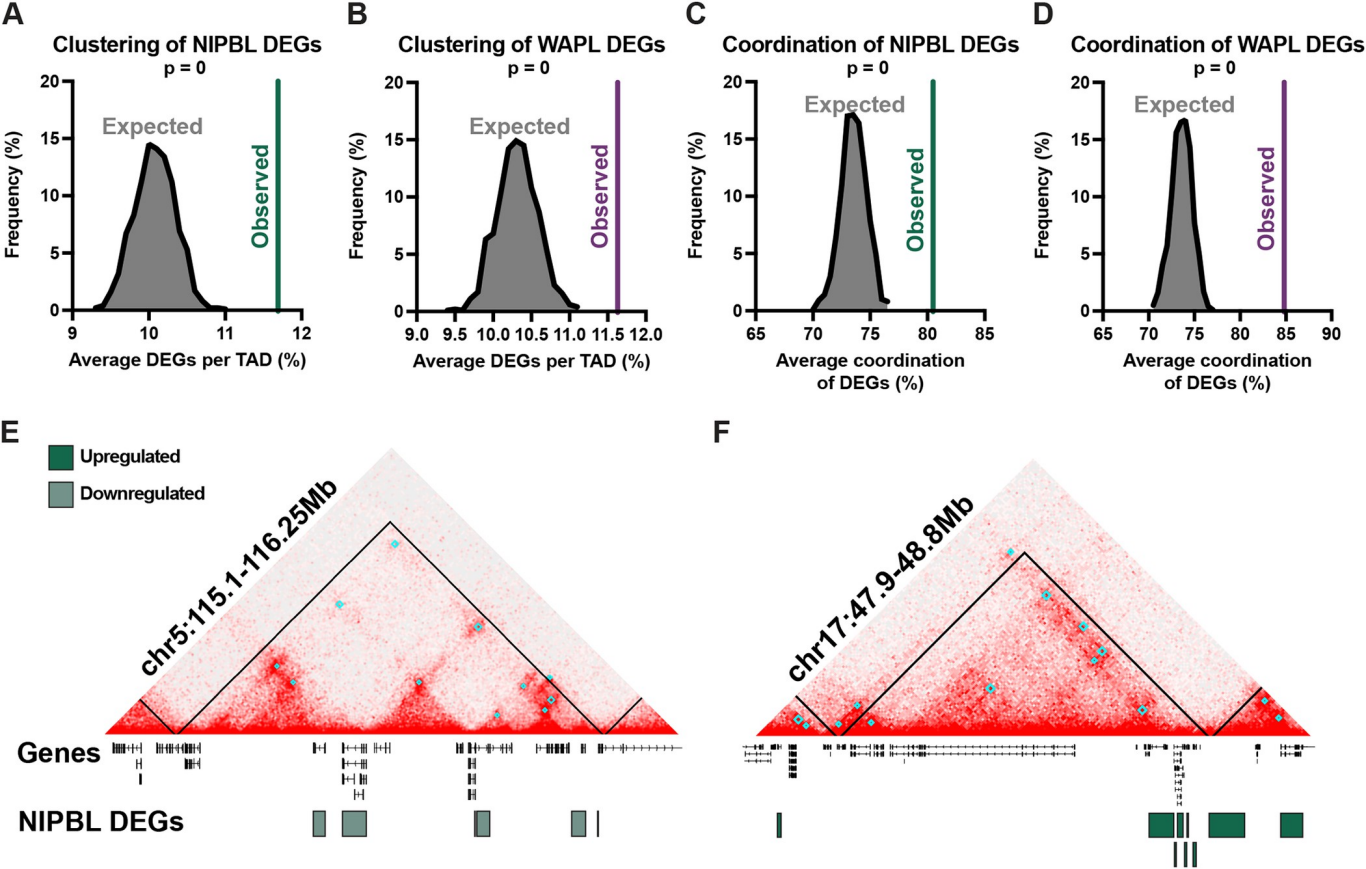
Cohesin-sensitive genes are clustered and coordinated within TADs. (A) The observed average percentage of NIPBL DEGs per TAD compared to a null distribution (expected). Permutations generated by shuffling the DEG and nonDEG designations across genes 1,000 times. Analysis limited to TADs with at least one expressed gene. Exact test, p = 0. (B) The observed average percentage of WAPL DEGs per TAD compared to a null distribution (expected). Permutations generated by shuffling the DEG and nonDEG designations across genes 1,000 times. Analysis limited to TADs with at least one expressed gene. Exact test, p = 0. (C) The average coordination of NIPBL DEGs compared to a null distribution generated by shuffling the fold change amongst the DEGs 1,000 times. Analysis limited to TADs with at least two expressed genes. Exact test, p = 0. (D) The average coordination of WAPL DEGs compared to a null distribution generated by shuffling the fold change amongst the DEGs 1,000 times. Analysis limited to TADs with at least two expressed genes. Exact test, p = 0. (E) Representative TAD with 100% DEG coordination on chr5 which contains six downregulated NIPBL DEGs. Black lines represent TADs, cyan boxes represent loops. (F) Representative TAD with 100% DEG coordination on chr17 which contains nine upregulated NIPBL DEGs. Black lines represent TADs, cyan boxes represent loops.

To further investigate the relationship between gene expression and chromatin topology, we asked whether genes differentially expressed after NIPBL or WAPL knockdown were arranged randomly throughout the genome or instead clustered within TADs as has been observed for active genes in general [21–23]. We computationally permutated the assignment of DEG or nonDEG to all active genes 1,000 times and found that both NIPBL- and WAPL-sensitive genes were clustered in TADs to a similar extent, albeit slightly more, to the expectation of active genes in general (Fig 3A and 3B).

We then investigated whether neighboring DEGs in each TAD have coordinated changes in their expression following cohesion dysfunction. For each TAD containing at least two DEGs, we calculated a coordination score based on the directionality of gene expression changes. We found that genes differentially expressed after NIPBL and WAPL knockdown were on average 80.5% and 84.8% coordinated, respectively, which was significantly greater than expected (Fig 3C and 3D). Moreover, we found that TADs with 90–100% coordination were significantly enriched above the null distribution, whereas TADs with 50–60% coordination were significantly depleted (S3A and S3B Fig). This suggests that DEGs are dysregulated in a coordinated fashion when they are found within the same TAD. Indeed, 52% and 60% of TADs with >2 DEGs had 100% coordination of genes differentially expressed after NIPBL and WAPL knockdown, respectively. This was especially apparent at a 1 Mb-sized TAD on the q arm of chromosome 5 that harbors six DEGs downregulated after NIPBL knockdown, two of which were also downregulated after WAPL knockdown (Fig 3E). Similarly, a TAD on the q arm of chromosome 17 harbored seven DEGs upregulated after NIPBL knockdown, five of which were also upregulated after WAPL knockdown (Fig 3F). In both examples, all DEGs were also enriched at loop anchors.

Considering the high coordination of DEGs within TADs, we reasoned that enhancer(s) within a domain might be activated or repressed after knockdown, and therefore affect the expression of all nearby genes. Alternatively, changes in the spatial organization of chromatin within a TAD might elicit miscommunication between regulatory elements and promoters separate from altered enhancer activity. To distinguish between these possibilities, we identified putative enhancer elements from the PRO-seq data using the discriminative Regulatory-Element detection algorithm (dREG). dREG peaks were further refined to predict 23,741 active enhancers in HCT116 cells. We then analyzed changes in PRO-seq signal at the dREG peaks to test whether eRNA synthesis, and thus enhancer activity, was changed in the knockdown conditions. We found that most (96%) enhancer peaks did not change after NIPBL or WAPL knockdown, suggesting that the changes in transcription were not due to altered enhancer activity (S3D and S3E Fig). Instead, these data, along with our FISH results, support a model in which changes in gene expression due to cohesin dysfunction are caused by changes in local chromatin topology at the level of loops and TADs.

### Co-depletion of NIPBL and WAPL restores TAD boundary strength by Oligopaint FISH

**Fig 4:**
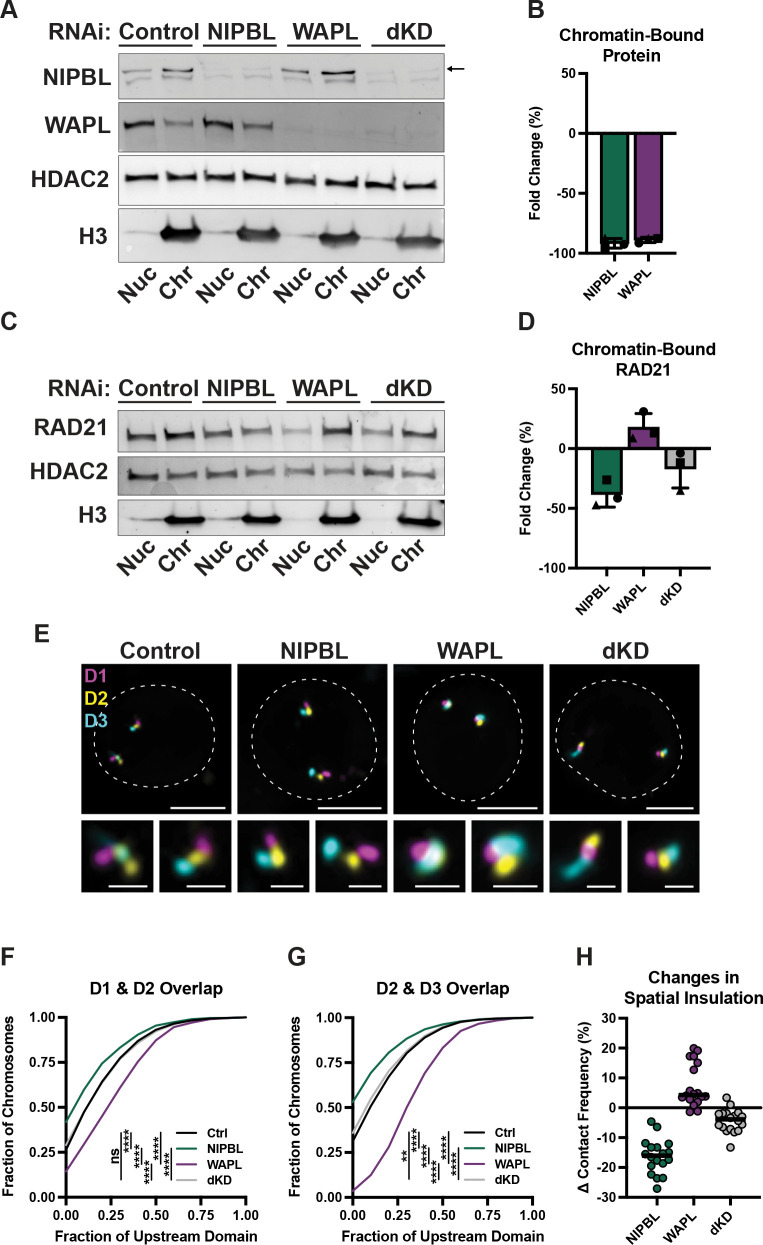
Co-depletion of NIPBL and WAPL restores TAD boundary strength by Oligopaint FISH. (A) Fluorescent western blot to NIPBL (top band, see arrow) and WAPL in nuclear (nuc) and chromatin-bound (chr) subcellular protein fractionations of RNAi control, NIPBL, WAPL, and double knockdown (dKD) depleted HCT116 cells. All bands are from the same blot with different exposures to optimize band detection. (B) Mean fold change (%) of NIPBL and WAPL bound to chromatin in the double knockdown condition. Each symbol represents a biological replicate, error bars represent standard deviation. (C) Fluorescent western blot to RAD21 in nuclear (nuc) and chromatin-bound (chr) subcellular protein fractionations of RNAi control and NIPBL and WAPL double knockdown (dKD) depleted HCT116 cells. All bands from the same blot. (D) Mean fold change (%) of RAD21 bound to chromatin in RNAi control, NIPBL, WAPL, and double knockdown (dKD) depleted HCT116 cells. Each symbol represents a biological replicate, error bars represent standard deviation. (E) Representative FISH images for three domains at chr2:217-222Mb in RNAi control, NIPBL, WAPL, and NIPBL and WAPL co-depleted HCT116 cells. Dashed line represents nuclear edge, scale bar, 5μm (above) or 1μm (below). (F) Cumulative frequency distribution of overlap between the neighboring domains D1 and D2 on chr2 in RNAi control (n = 2,172 chromosomes), NIPBL (n = 1,514 chromosomes), WAPL (n = 1,704 chromosomes), or dKD (n = 1,620 chromosomes) depleted HCT116 cells. Two-tailed Mann-Whitney test, **** p < 0.0001, ns = not significant (p = 0.79). (G) Cumulative frequency distribution of overlap between the neighboring domains D2 and D3 on chr2 in RNAi control (n = 2,188 chromosomes), NIPBL (n = 1,571 chromosomes), WAPL (n = 1,719 chromosomes), or dKD (n = 1,661 chromosomes) depleted HCT116 cells. Two-tailed Mann-Whitney test, **** p < 0.0001, ** p = 0.0014. (H) Change in contact frequency across 18 domain pairs in HCT116 cells depleted for NIPBL, WAPL, or both. Each dot represents the median of ≥ 4 biological replicates at each locus.

Haarhuis et al. previously showed that knockout of both the NIPBL co-factor MAU2 and WAPL restored normal chromatin looping across the genome as measured by Hi-C [2]. This motivated us to test whether co-depletion of NIPBL and WAPL could also balance one another. We simultaneously knocked down both proteins for 72 hours, resulting in 96% and 94% chromatin depletion, respectively, similar to the single knockdown conditions (Fig 4A and 4B). Importantly, cell growth, mitotic entry, and chromosome segregation remained unaltered in the double knockdown condition, indicating that HCT116 cells can tolerate simultaneous depletion of both proteins across a minimum of four divisions (S4A–S4D Fig). We first measured cohesin levels after subcellular fractionation, which demonstrated a partial rescue of RAD21 levels on chromatin compared to the single knockdowns (Figs 1D, 4C and 4D). We next performed Oligopaint FISH to assess boundary strength in single cells as measured by the amount and frequency of spatial overlap between neighboring domains. Despite only partial rescue of chromatin-bound cohesin levels, the double knockdown restored the distribution of spatial overlap across two boundaries analyzed on chromosome 2 (Figs 4E–4G, S4E and S4F). Using HiDRO, we extended this assay to sixteen additional loci across the genome and found that all but one boundary showed partial or complete rescue of inter-domain interactions after double knockdown of NIPBL and WAPL (Figs 4H and S4G). This further supports findings reported by Haarhuis et al. [2], in that balancing levels of pro- and anti-cohesin factors have the capacity to complement one another and extends this finding to single cells. What remained unknown is whether rescue of chromatin misfolding is sufficient to restore normal gene expression across the genome, which we address below.

### NIPBL and WAPL balance cohesin activity to regulate gene expression

**Fig 5:**
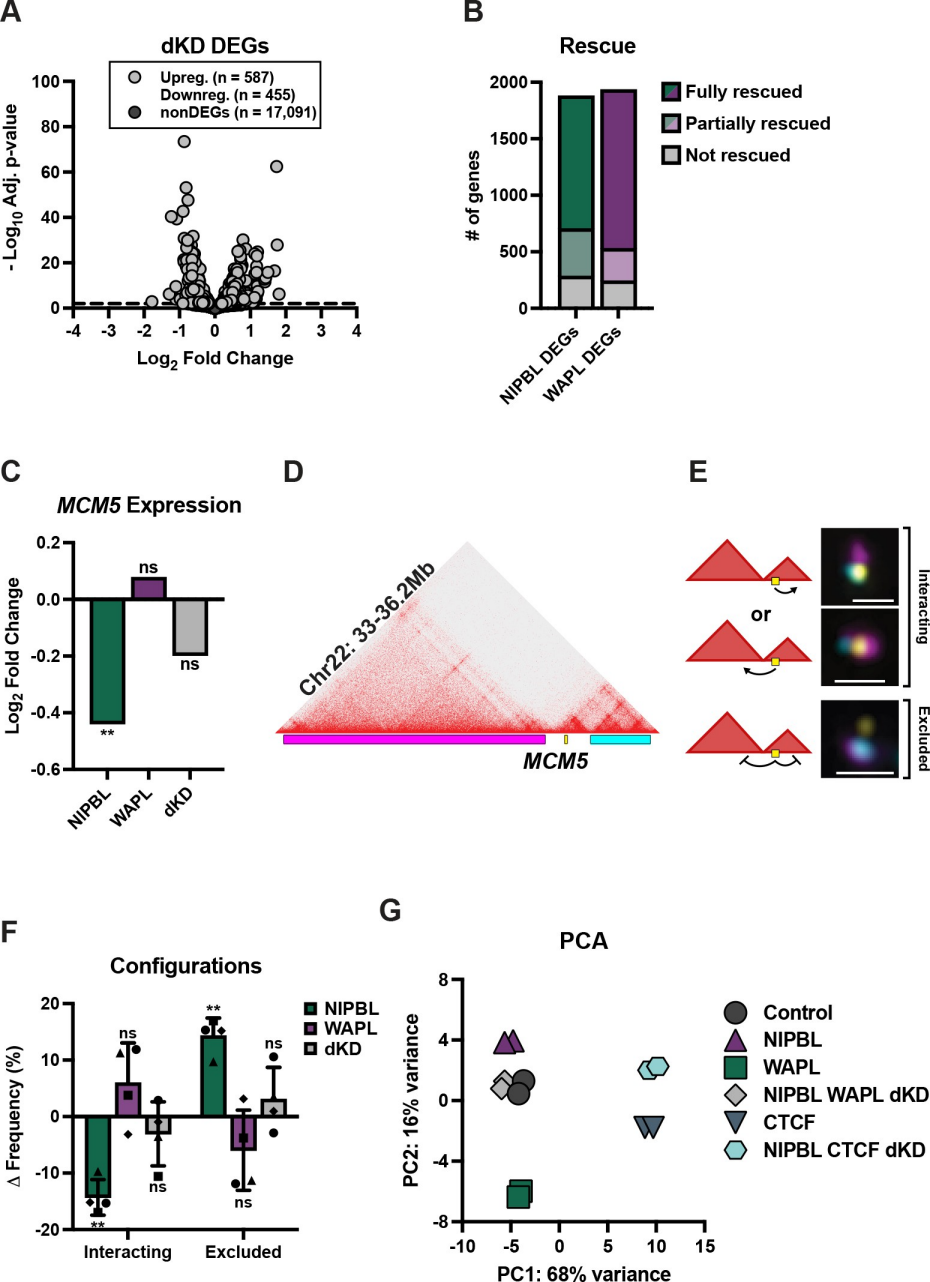
NIPBL and WAPL balance cohesin activity to regulate gene expression. (A) The log_2_(fold change) of genes after NIPBL and WAPL double knockdown versus their significance. DEGs are in light grey (587 up, 455 down) and non-significantly changed genes (adjusted p-value > 0.01) are in dark grey. (B) Number of NIPBL and WAPL DEGs fully, partially, or not rescued in the double knockdown condition. (C) The log_2_(fold change) of *MCM5* expression in the NIPBL, WAPL, or double knockdown conditions. ** p = 0.002, ns = not significant (p = 0.58 for WAPL, p = 0.13 for dKD). (D) Oligopaint design to *MCM5* and neighboring domains at chr22:32–36.2Mb. (E) Cartoon diagrams and representative FISH images of the possible interactions between *MCM5* and its neighboring domains at chr22:32–36.2Mb. The “interacting” configuration is defined as the majority of the *MCM5* signal overlapping either the up or downstream domain. “Exclusion” is defined as the majority of *MCM5* signal non-overlapping with either neighboring domain. Dashed line represents nuclear edge, scale bar 1μm. (F) Change in the frequency of interacting and exclusion between *MCM5* and neighboring domains at chr22:32–36.2Mb in RNAi control, NIPBL, WAPL, or double knockdown cells. Each bar represents the mean of four biological replicates, error bars represent standard deviation. Two-tailed paired t-test, ** p = 0.003 interacting; p = 0.003 exclusion, ns = not significant (p ≥ 0.19). (G) Principal component analysis plot of the PRO-seq data. Each symbol represents one biological replicate of knockdown.

We performed PRO-seq in cells co-depleted of NIPBL and WAPL to assess changes in the nascent transcriptome (Fig 5A). We compared the DEGs in the NIPBL knockdown and double knockdown conditions to determine which, if any, gene expression changes were rescued by co-depletion with WAPL. We found that 1,174 of the 1,877 (62.5%) DEGs identified in the NIPBL knockdown were completely rescued and were no longer significantly differentially expressed in the double knockdown (Fig 5B). This included *MCM5*, which is a boundary-proximal gene found to be sensitive to loss of either NIPBL (this study) or RAD21 [15,19] (Fig 5C). To examine whether the double knockdown might rescue interactions between MCM5 and its cis-regulatory domains, we repeated a FISH experiment previously designed to measure interactions between the gene and its neighboring domains [19] (Fig 5D and 5E). Indeed, co-depletion of NIPBL and WAPL restored the distribution of *MCM5*-domain configurations to that observed in the control samples (Fig 5F). Thus, restoring chromatin folding patterns at this locus was accompanied by a rescue of *MCM5* gene expression.

An additional 421 NIPBL-sensitive genes were partially rescued by WAPL co-depletion; these genes were still significantly misexpressed in the same direction as in the NIPBL single knockdown, but their fold change was diminished (Fig 5B). Collectively, co-depletion of NIPBL and WAPL fully or partially rescued 85% of the genes differentially expressed after NIPBL knockdown (Fig 5B). These rescued genes included both unique and shared DEGs from either condition, were equally up- and downregulated (841 upregulated and 754 downregulated) and were enriched in the same top six GO terms as the genes differentially expressed after NIPBL knockdown (S1 and S4 Tables), suggesting that the major biological processes disrupted by NIPBL depletion can be rescued by double knockdown with WAPL.

Given the large number of uniquely sensitive genes between NIPBL and WAPL, we next reciprocally examined whether WAPL-dependent gene expression might also be rescued by co-depletion of NIPBL. Remarkably, of the 1,932 genes sensitive to WAPL depletion, 1,405 were fully rescued and another 287 were partially rescued in the double knockdown condition (Fig 5B). In total, expression of 88% of genes differentially expressed after WAPL depletion was restored by co-depletion of NIPBL. Rescued DEGs represented both up- and down-regulated genes (769 and 923, respectively), and were enriched in similar biological processes to those genes differentially expressed after WAPL single knockdown (S3 and S5 Tables). Taken together, these data show that co-depletion of NIPBL and WAPL can complement each other and correct for the majority of gene misexpression observed in either single depletion.

### CTCF loss partially rescues gene misexpression in NIPBL-depleted cells

Considering WAPL co-depletion with NIPBL could restore gene expression to normal levels, we next asked whether any opposing regulator of cohesin activity might have this capacity. Therefore, we next investigated whether co-depletion of CTCF, which inhibits loop extrusion by stabilizing cohesin on chromatin [24–26], might have a similar effect to that of WAPL depletion. CTCF knockdown alone significantly altered the expression of 3,889 genes (S5A Fig). As previously observed in other cell types, the majority of CTCF DEGs (92%) had modest fold changes (<two-fold change; S5A Fig) [13,14,18,27–30]. Less than 22% of these genes were also sensitive to NIPBL or WAPL depletion, suggesting that the effect of CTCF knockdown on transcription was mostly distinct from that of cohesin dysregulation (S5B Fig). However, of the 1,877 genes differentially expressed after NIPBL knockdown, 959 were fully rescued by co-depletion of CTCF (S5C Fig). Another 280 genes showed decreased changes in expression; therefore, a total of 66% of DEGs after NIPBL depletion were partially or fully rescued in the double knockdown condition (S5C Fig). Interestingly, 85% of the genes rescued by CTCF depletion were also rescued by WAPL depletion, consistent with their shared role in restricting chromatin loop extrusion.

To simultaneously compare all gene expression changes across the six conditions and two biological replicates each, we performed a principal component analysis (PCA) of the PRO-seq datasets (Fig 5G). This analysis reiterates our finding that NIPBL and WAPL depletion had opposing effects on gene expression; these two conditions separated along the second principal component. All replicates for control and NIPBL-WAPL double knockdown conditions were clustered strikingly close to one another, reflecting the genome-wide restoration of transcription observed in these samples. Replicates involving CTCF knockdown were distinctly separated from the other samples along the first principal component, consistent with a large effect on different genes; however, we noted that CTCF samples trended along the second principal component toward samples with depletion of WAPL. Finally, co-depletion with NIPBL did not affect the variance of the first principal component; however, the second principal component reflected the partial rescue of gene expression across all samples. Together, these data strongly support the notion that reduced cohesin activity via NIPBL depletion can be functionally offset by removal of either its negative regulator (WAPL) or the physical barriers (CTCF) that restrict loop-extrusion events.

## Discussion

The roles of NIPBL and WAPL in gene expression have been extensively studied in independent contexts. Here, we depleted each in the same cell type, permitting direct comparative analysis between the two factors. In this study, we reduced the levels of cohesin regulators NIPBL and WAPL in human HCT116 cells to alter chromatin folding and performed nascent transcriptional analysis on each. Given that NIPBL and WAPL are opposing regulators of cohesin [2,11,12,16], one prediction might be that each of their knockdowns would alter the same set of genes but in opposite directions. Instead, we found that most (~70%) DEGs were exclusive to either knockdown condition. Moreover, the 30% of DEGs that were shared between the knockdowns tended to be differentially expressed in the same direction, similar to what has been reported following acute degradation of RAD21 or WAPL in mESCs [31]. In general, DEGs were enriched at cohesin binding sites and chromatin loops, consistent with their dysregulation due to aberrant looping. Indeed, we found that NIPBL- and WAPL-sensitive genes found in the same TAD are significantly and coordinately up- or downregulated.

**Fig 6:**
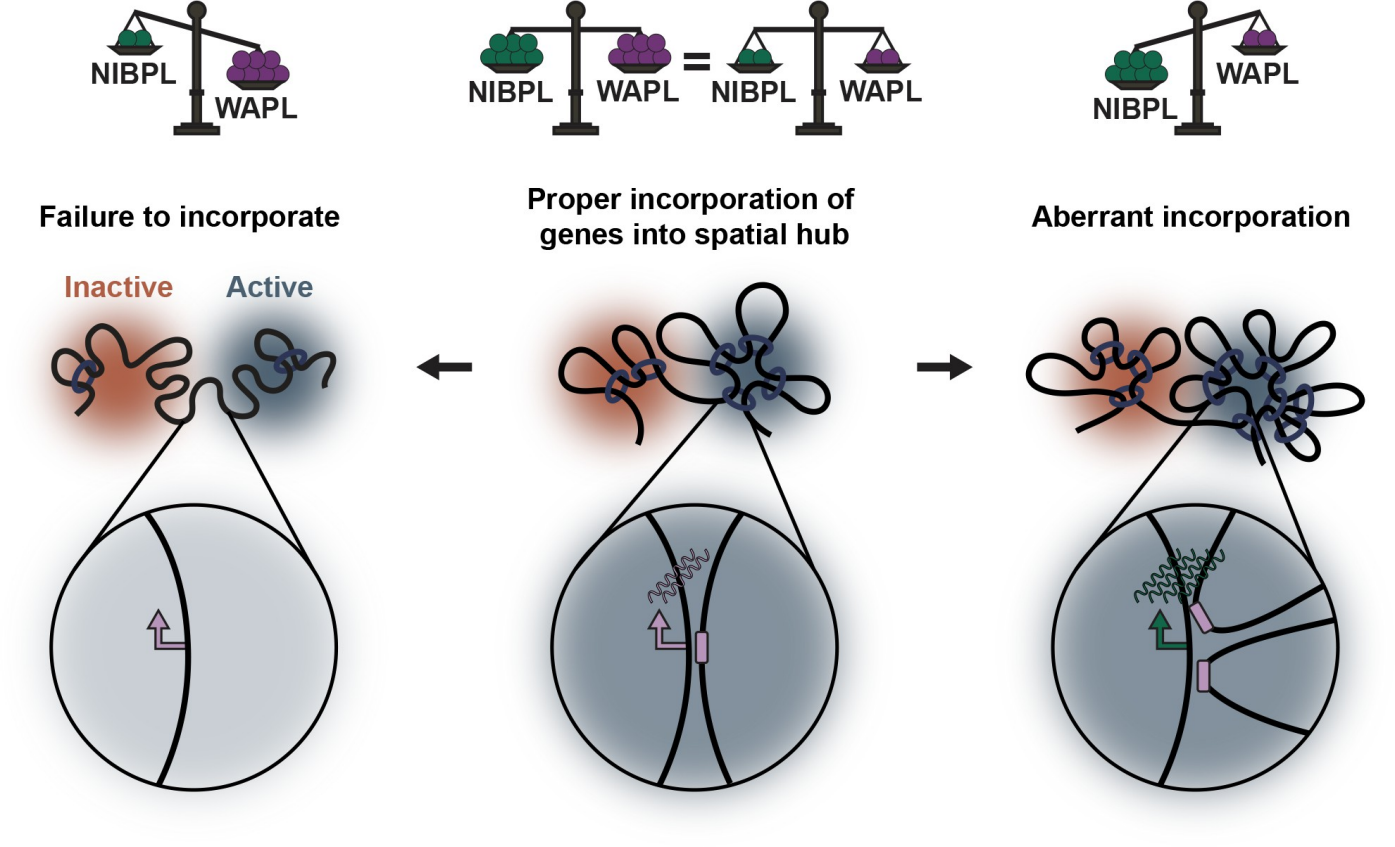
NIPBL and WAPL balance cohesin activity to regulate chromatin organization and gene expression. Cohesin normally promotes the compaction and clustering of chromatin into TADs, which we propose act as regulatory hubs. These hubs can either stimulate (Active, blue) or repress (Inactive, Red) expression of nearby genes in a dynamic fashion. Depletion of NIPBL would limit loop extrusion events and processivity, resulting in less frequent incorporation of distal genes into their target hubs. As depicted, an active gene (purple) could be displaced from its active hub and spatially separated from its target enhancer, leading to its downregulation. In contrast, WAPL depletion would effectively increase loop extrusion events and lead to the ectopic incorporation of distal genes into new hub environments. As depicted, a silent gene (green) could be aberrantly activated when positioned inside an active hub with active enhancers (purple) in close proximity. Co-depletion of NIPBL and WAPL would therefore balance cohesin activity, restore chromatin folding, and correct hub formation to effectively rescue gene expression.

Our results are consistent with a model in which genomic regions are co-regulated within spatial hubs. These hubs could either promote or repress transcription, depending on the local environment (Fig 6) [32–41]. When NIPBL is depleted, loop extrusion is limited; consequently, distal chromatin may not reach their target regulatory hubs as efficiently, resulting in altered expression of several nearby genes. This is consistent with our analysis of the *MCM5* locus, in which the downregulated gene is displaced from neighboring domains following loss of cohesin [19]. The opposite would occur in the absence of WAPL, with regions beyond those normally incorporated into hubs brought into closer proximity, providing an explanation for its role in expression of a different set of genes. Importantly, we found no change to eRNA levels following NIPBL or WAPL depletion. Therefore, while not essential for gene expression, NIPBL and WAPL may instead function to balance the exposure of promoters within a TAD to local gradients of eRNAs and activated TFs [42].

Interestingly, balancing the expression of these two ubiquitously expressed and essential proteins rescued the effects of knockdown of either single protein. In total, ~85% of genes differentially expressed after NIPBL or WAPL knockdown were at least partially rescued by simultaneous knockdown of both proteins to ~10% control levels. Co-depletion also partially restored the levels of chromatin-bound cohesin and rescued the spatial insulation between TADs at the single-allele level by FISH. Contact between a boundary-proximal gene sensitive to cohesin loss, *MCM5*, and its neighboring regulatory domain, was also rescued in the double knockdown condition. This was accompanied by a correction of *MCM5* expression, which is consistent with its dependency on proper cohesin activity. Indeed, we found that co-depletion of NIPBL and CTCF largely rescued the same DEGs as WAPL. This further supports the notion that proper gene expression is achieved by balancing the regulators of cohesin-mediated loop extrusion.

Together, our data are in full agreement with several intriguing findings in which co-depletion of WAPL and NIPBL or MAU2 functionally restore proper organismal development, cellular differentiation rates, or cell viability across *Drosophila*, mouse, and human systems [2,43,44]. In this study, we show that this rescue extends to the molecular level resulting in near complete complementation of gene expression changes across the entire genome. Excitingly, while our manuscript was under review, a preprint describing a similar partial rescue of transcription was observed when the dosage of both NIPBL and WAPL are decreased in embryonic mouse brains [45]. We therefore propose that the correct ratio, rather than the absolute amount, of NIPBL and WAPL is necessary to properly modulate cohesin activity, organize chromatin, and regulate transcription.

## Materials and methods

### Cell culture

HCT116 cells were obtained from AATC (ATCC CCL-247 Colon Carcinoma; Human; Lot 70009735) and HCT116-RAD21-AID cells were obtained from Natsume et al. [46]. Cells were cultured in McCoy’s 5A media supplemented with 10% FBS, 2 mM L-glutamine, 100 U/ml penicillin, and 100ug/ml streptomycin and filtered using a 0.22-μm PES membrane at 37°C with 5% CO_2_. HCT116-RAD21-AID cells were re-selected with 100μg/ml G418 and 100μg/ml HygroGold prior to experiments. Prior to FISH on slides, HCT116-RAD21-AID cells were synchronized as previously described in [19].

### RNAi

The following siRNAs (Dharmacon) were used: Non-targeting control (D-001210-05-05), NIPBL (J-012980-08; target sequence: 5’-CAACAGAUCACAUAGAGUU-3’), WAPL (L-026287-01-0005; target sequences administered as a pool: 5’-GGAGUAUAGUGCUCGGAAU-3’, 5’-GAGAGAUGUUUACGAGUUU-3’, 5’-CAAACAGUGAAUCGAGUAA-3’, 5’-CCAAAGAUACACGGGAUUA-3’), and CTCF (L-020165-00-0010; target sequences administered as a pool: 5’-GAUGAAGACUGAAGUAAUG-3’, 5’-GGAGAAACGAAGAAGAGUA-3’, 5’-GAAGAUGCCUGCCACUUAC-3’, 5’-GAACAGCCCAUAAACAUAG-3’). siRNAs were incubated for 20 minutes at room temperature with RNAiMAX Lipofectamine transfection reagent (ThermoFisher) in Opti-MEM reduced serum media (ThermoFisher) and seeded into wells. HCT116 cells were trypsinized and resuspended in antibiotic free media (McCoy’s 5A media supplemented with 10% FBS and 2 mM L-glutamine), then plated onto siRNA for a final siRNA concentration of 50 nM (non-targetting control and WAPL), 100 nM (NIPBL), or 150 nM (CTCF). For CTCF knockdowns, cells were retreated with 150nM CTCF siRNAs 24 hours after initial treatment. After 72h (NIPBL, WAPL, non-targeting control) or 96h from the initial RNAi treatment (CTCF), cells were harvested for experiments.

### Western blotting

To prepare samples, cells were trypsinized and resuspended in fresh media, washed once in cold Dulbecco’s PBS, and then centrifuged at 500g for 5 min at 4°C. Subcellular protein fractionations were performed using the Subcellular Protein Fractionation Kit for Cultured Cells (Thermo Scientific, Catalog no: 78840) according to the product manual. We used reagent volumes corresponding to 10μl packed cell volume for 4x10^6^ cells. In step 10, we incubated samples at room temperature for 15 minutes. To extract the whole cell lysate (WCL), samples were resuspended in 1x RIPA buffer with fresh protease inhibitors (200μl per 5x10^6^ cells), nutated 30 min at 4°C, centrifuged at 16,000g for 20 min at 4°C. Supernatant was extracted and stored at -80°C. The Pierce BCA protein assay kit (Catalog no. 23225) was used to quantify the amount of protein per sample.

For each sample, 12–15μg protein was combined with NuPAGE LDS sample buffer and sample reducing agent (Thermo Fisher Scientific). Samples were denatured at 70°C for 10 min, then cooled on ice. Benzonase was added to the WCL samples (0.5μl), followed by a 15-min incubation at 37°C. We 25μl of each sample on Mini-PROTEAN TGX precast gels (Bio-Rad, catalog no. 456–1083) at 35mA. Protein was then transferred to 0.2 μm nitrocellulose membrane at 110 V for 1hr. The nitrocellulose membrane was then washed twice in TBS (150 nM NaCl, 20 mM Tris) for 5 min, and blocked in 5% milk in TBS-T (TBS with 0.05% Tween 20) for 30 min. The membrane was washed again twice in TBS-T, then incubated with primary antibody diluted in 5% milk in TBS-T overnight at 4°C. The following day, the nitrocellulose membrane was washed twice in TBS-T for 5 min each wash, then incubated with secondary antibodies diluted in 5% milk in TBS-T for 1 h at RT. The nitrocellulose filter was then washed twice in TBS-T for 15 min each wash, followed by a final 15-min wash in TBS. For blots probed with secondary antibodies conjugated to horseradish peroxidase (HRP), the membrane was incubated in a 1:1 mixture of Clarity Western ECL Substrate reagents (Bio-Rad). Blots were then imaged on a ChemiDoc MP Imaging System and analyzed with Bio-Rad Image Lab software (v6.1.0 build 7).

The following primary antibodies were used: NIPBL (sc-374625; 1:400), WAPL (Cell Signaling Technology (CST) D9J1U; 1:1,000), RAD21 (ab992, 1:1,000), HDAC2 (Cell Signaling Technology 5113S, 1:2,000), GAPDH (CST 5174S, 1:2,000), H3 (ab1791, 1:4,000). The following secondary antibodies were used: anti-mouse IgG, HRP-linked Antibody (CST #7076; 1,5,000), anti-rabbit IgG, HRP-linked antibody (CST #7074; 1,5,000), Cy3 AffiniPure Goat Anti-Rabbit IgG (Jackson ImmunoResearch 111-165-003, 3:20,000), IRDye 800CW Goat anti-Mouse IgG Secondary Antibody (LI-COR, 3:10,000).

### RNA extraction and RT-qPCR

HCT116 derived RNA was isolated using the RNeasy Plus Kit (Qiagen) according to manufacturer’s instructions. For complementary DNA (cDNA) synthesis, a 50μl reaction containing 20μl RNA, 1500pmol Oligo dT primer (IDT), 1.6mM dNTPs, 1x RT Buffer (Thermo Scientific), 0.5μl RNase OUT (Invitrogen), and 0.7μl Maxima RT (Thermo Scientific) was incubated at 50°C for two hours then at 85°C for 5 min. Samples were stored at -20°C until use. RT-PCR was performed using PowerUP Sybr (ThermoFisher, #A25741) based on manufacturer’s instructions. Briefly, cDNA was diluted to a working concentration of 6μg and HCT116 genomic DNA (gDNA) was diluted in a 1:10 serial dilution. A 6μl reaction was prepared per well, with 1x PowerUP Sybr and 0.2μM of the forward and reverse primers and combined with 4μl diluted DNA. Each reaction was performed in triplicate. qPCR was performed on the QuantStudio7 Flex System. YWHAZ and TBP were used as reference control genes. The sequences of oligonucleotides used for qPCR are: NIPBL forward primer: 5’-TCTCTTTGTTACTTGTCTGTTTCC-3’ and reverse primer 5’-ATGTTTTGCTTTGAAAACCAGTG-3’; WAPL forward primer 5’-GAACTAAAACAGCTCCATCACC-3’ and reverse primer 5’-CACACTTTCAGGCACACCAG-3’; YWHAZ forward primer 5’-CCCGTTTCCGAGCCATAAAAG-3’ and reverse primer 5’-TTTGGCCTTCTGAACCAGCTC-3’; and TBP forward primer 5’-ACAGCTCTTCCACTCACAGAC-3’ and reverse primer 5’-ATGGGGGAGGGATACAGTGG-3’.

### FISH probe design & synthesis

Oligopaint probes were designed as previously described [19]. Briefly, we designed probes to domains and subdomains based on ChIP-Seq and Hi-C data using the OligoMiner design pipeline [47]. Probe coordinates and details are listed in S6 Table. Oligopaints were designed to have either 80 bases of homology and were purchased from Twist Bioscience. Additional bridge probes were designed to the *MCM5* gene probe to amplify its signal [48]. Oligopaints were synthesized as previously described [19] with some modifications to allow for direct conjugation to fluorescent dyes. Specifically, aminoallyl-dUTP (ThermoFisher Scientific) was incorporated into the probes to allow for conjugation with Alexa 488 (ThermoFisher Scientific), Cy3 (Gold Biotechnology), or Alexa 647 (ThermoFisher Scientific).

### DNA fluorescence in situ hybridization (FISH)

#### FISH on slides

FISH was performed on slides to chr2: 217-222Mb (Figs 1J–1L and S1G–S1I) and chr22: 33–36.2Mb (Fig 4E–4G and 4I–4K). Cells were settled on poly(L-lysine)-treated glass slides for 2 h. Cells were then fixed to the slide or coverslip for 10 min with 4% formaldehyde in phosphate-buffered saline (PBS) with 0.1% Tween 20, followed by three washes in PBS for 5 min each wash. Slides and coverslips were stored in PBS at 4°C until use. Prior to FISH, slides were warmed to room temperature (RT) in PBS for 10 min. Cells were permeabilized in 0.5% Triton-PBS for 15 min. Cells were then dehydrated in an ethanol row, consisting of 2-min consecutive incubations in 70%, 90% and 100% ethanol. The slides were then allowed to dry for about 2 min at RT. Slides were incubated for 5 min each in 2xSSCT (0.3 M NaCl, 0.03 M sodium citrate and 0.1% Tween 20) and 2xSSCT/50% formamide at RT, followed by a 1-h incubation in 2xSSCT/50% formamide at 37°C. Hybridization buffer containing primary Oligopaint probes, hybridization buffer (10% dextran sulfate, 2xSSCT, 50% formamide and 4% polyvinylsulfonic acid (PVSA)), 5.6 mM dNTPs and 10 μg RNase A was added to slides, covered with a coverslip, and sealed with rubber cement. 50 pmol of probe was used per 25 μl hybridization buffer. Slides were then denatured on a heat block in a water bath set to 80°C for 30 min, then transferred to a humidified chamber and incubated overnight at 37°C. The following day, the coverslips were removed and slides were washed in 2xSSCT at 60°C for 15 min, 2xSSCT at RT for 10 min, and 0.2xSSC at RT for 10 min. Next, hybridization buffer (10% dextran sulfate, 2xSSCT, 10% formamide and 4% PVSA) containing secondary probes conjugated to fluorophores (10pmol per 25 μl buffer) was added to slides, covered with a coverslip and sealed with rubber cement. Slides were placed in a humidified chamber and incubated for 2 h at RT. Slides were washed in 2xSSCT at 60°C for 15 min, 2xSSCT at RT for 10 min, and 0.2xSSC at RT for 10 min. To stain DNA, slides were washed with Hoechst (1:10,000 in 2xSSC) for 5 min. Slides were then mounted in SlowFade Gold Antifade (Invitrogen).

Images were acquired on a Leica widefield fluorescence microscope, using a 1.4 NA ×63 oil-immersion objective (Leica) and Andor iXonμltra emCCD camera. All images were deconvolved with Huygens Essential v20.04.03 (Scientific Volume Imaging), using the CMLE algorithm, with a signal to noise ratio of either 40, and 40 iterations (DNA FISH) or signal to noise ratio of 40 and 2 iterations (DNA stain). The deconvolved images were segmented and measured using a modified version of the TANGO 3D-segmentation plug-in for ImageJ [49–51]. Edges of nuclei and FISH signals were segmented using a Hysteresis-based algorithm.

#### High-throughput DNA or RNA Oligopaints (HiDRO)

All other FISH experiments (Figs 1M, S1L, S1M, 4H and S4G**)** were performed using HiDRO as described in detail in [20]. All spins were performed at 1200 rpm for 2 min at RT unless otherwise indicated. When possible, automatic pipetting was performed by a Matrix WellMate (Thermo Fisher Scientific). For experiments in the HCT116-RAD21-AID cell line, 7.5x10^4^ cells in supplemented McCoy’s 5A media -/+ 500 μM auxin were seeded in 384-well plates (Perkin Elmer 6057300) and incubated at 37°C for 6 h. For RNAi experiments in the HCT116 cell line, plates were seeded with siRNA (see RNAi section for details) diluted in Opti-MEM reduced serum medium to a final concentration of 25nM per well. Plates were then spun and incubated at RT for 20 min. HCT116 cells were trypsinized and resuspended in antibiotic-free medium, then 2.5x10^3^ cells were seeded in each well. Plates were spun and incubated at 37°C for 72 h.

Following incubation, media was aspirated, all wells had PBS added to them, and plates were spun. PBS was aspirated and cells were fixed in each well with 4% PFA, 0.1% Tween-20 in 1x PBS for 10 minutes at RT. Plates were spun once during fixation. Then plates were rinsed with 1xPBS and washed twice for 5 minutes with 1xPBS with a spin during each wash. 70% ethanol was then added to each well, plates were sealed with foil plate covers (Corning) and stored for at least 20 hours at 4°C until used for FISH.

On the first day of DNA FISH, ethanol was aspirated and plates were washed in 1xPBS for 10 min to reach RT. Plates were then spun, washed briefly again in 1xPBS and spun again. Cells were permeabilized for 15 min in 0.5% Triton-X and 5 minutes in 2xSSCT (0.3 M NaCl, 0.03 M sodium citrate and 0.1% Tween 20). Then 2xSSCT/50% formamide was added to all wells, and plates were double sealed with foil covers. Pre-denaturation was performed at 91°C for 3 min and then 60°C for 20 min on heat blocks (VWR). After plates were spun, foil covers were removed and hybridization mix was added to wells. Hybridization mix consisted of 50% formamide, 10% dextran sulfate, 4% PVSA, 0.1% Tween-20, 2xSSC, and each probe at 0.1pmol/μl. 2pmol of probe was used per 20μl of hybridization mix. Hybridization mix was viscous and thus pipetted using a manual multichannel pipette. After spinning, plates were double sealed with foil covers and denatured at 91°C for 20 min on heat blocks. Heat blocks were covered to block light and preserve primary fluorescently labeled probes. Plates were spun after denaturation and then hybridized overnight at 37°C.

The following day, hybridization mix was aspirated, and plates were washed quickly twice with RT 2x SSCT, then with 60°C 2xSSCT for 5 min. Plates were then washed with RT 2x SSCT for 5 min. Nuclei were stained by washing for 5 min in Hoescht (1:10,000 in 2x SSCT). Plates were spun, washed for 15 min with 2x SSC and spun again. Finally, plates were mounted with imaging buffer (2x SSC, 10% glucose, 10mM Tris-HCl, 0.1 mg/ml catalase, 0.37 mg/ml glucose oxidase) and imaged within 5 days of FISH.

Images for HiDRO experiments were acquired on a Molecular Devices Image Xpress Micro 4 Confocal high-content microscope with 0.42 um pinhole and 1.4 NA 60X water immersion objective (Molecular Devices). Max projections of z-series (6 images, 0.5 uM spacing) were generated automatically in MetaXpress and used for downstream analyses. Contact between domains was defined by > 250 nm signal colocalization.

### Hi-C analysis

Hi-C library from parental HCT116 cells were generated from 1x106 cells using the Arima-HiC+ kit (Arima Genomics) and the Kapa Hyper Prep Kit with KAPA Library Amplification Primer Mix (KK8502), according to manufacturer’s recommendations. Libraries were validated for quality and size distribution using the Qubit dsDNA HS Assay Kit (Invitrogen, cat# Q32851), KAPA Library Quantification kit (Roche, Cat# KK4824) and TapeStation 2200 (Agilent). Hi-C libraries were paired-end sequenced (61bp+61bp) on a NovaSeq 6000 (Illumina). Raw reads were processed with HiC-Pro (version 2.11.1) to obtain putative interactions with default parameters except LIGATION_SITE = GATCGATC and GENOME_FRAGMENT generated for MboI restriction enzyme (Servant et al., 2015). For downstream analyses, ValidPairs were converted to hic files using the “hicpro2juicebox.sh” in utils of HiC-Pro. We recovered 507,359,205 contacts.

We called chromatin loops using the HICCUPS tool in Juicer (version 1.22.01) using the same settings as [52] for high resolution maps, as shown here: "-k KR -f .1,.1 -p 4,2 -i 7,5 -t 0.02,1.5,1.75,2 -d 20000,20000". TAD were called using the hicFindTAD function of the HiCExplorer package (version 3.7.2) [53–55]. First,.hic files were first converted to.cool files at 50 kb resolution using hic2cool (https://github.com/4dn-dcic/hic2cool) and then corrected used the "cooler balance" function from the cooltools package (https://github.com/open2c/cooler) [56] (Abdennur and Mirny, 2020). These.cool files were then converted to.h5 format using "hicConvertFormat" from HiCExplorer package, and the resulting.h5 files were used to call TADs with the following parameters of hicFindTADs: "—correctForMultipleTesting fdr—minBoundaryDistance 100000—delta 0.4".

### Permutation analyses

Permutation analysis was used to create an "expected" null distribution with which to compare the observed clustering and coordination of DEGs. Most (95%) active genes were within a called TAD. For clustering, all genes in the genome were either assigned transcription status (active/non-active) or the DEG status (DEG/nonDEG). Observed clustering was calculated by measuring the percentage of active genes/DEGs per TAD and comparing it against a 1000 random permutations, where the transcription/DEG status was shuffled across all genes for each permutation while keeping the number of genes in each category constant. A p-value was reported as the percentile ranking of the observed clustering against this permutation distribution. For analysis of the coordination of DEGs within TADs, the same approach was taken as above, with each DEG assigned a direction of misexpression (up/down) and the observed coordination across TADs compared against 1000 random permutations.

### PRO-seq & analysis

#### Cell permeabilization

RNAi was performed as previously described. Following 72 h knockdown, cells were rinsed with Dulbecco’s phosphate buffered saline (DPBS) and treated with trypsin to detach them from the plate. Cells were resuspended in cold supplemented McCoy’s 5A media and three wells of a 6-well plate were pooled per replicate and placed on ice. From this point on, all steps were performed on ice, all buffers were pre-chilled, and samples were spun at 300xg for 10 min at 4°C, unless otherwise noted. Cells were rinsed in PBS containing 1% Bovine Serum Albumin (BSA) to prevent cell clumping, and then resuspended in 1 ml Buffer W (10 mM Tris-HCl pH 8.0, 10 mM KCl, 250 mM sucrose, 5 mM MgCl2, 0.5 mM DTT, 10% glycerol, 1% BSA) then strained through a 35 μm nylon mesh filter. The tube was rinsed with an additional 1ml of Buffer W and passed through the same strainer. A 9X volume of Buffer P (Buffer W + 0.1% IGEPAL CA-630) was immediately added to each sample and nutated for 2 minutes at room temperature. Cells were resuspended in 500μl Buffer F (50 mM Tris-Cl pH 8.3, 40% glycerol, 5 mM MgCl2, 0.5 mM DTT, 1 μL/ml SUPERaseIn RNase inhibitor, 0.5% BSA) using a wide-bore P1000 tip and transferred to a low binding tube. The original tube was rinsed with another 500μl Buffer F and the samples were pooled. Samples were spun at 400xg and resuspended to 5 x 10^6^ cells in 500μl Buffer F. Samples were flash frozen in liquid nitrogen and stored at −80°C

#### PRO-seq library construction

PRO-seq library construction and data analysis was performed by the Nascent Transcriptomics Core at Harvard Medical School, Boston, MA. Aliquots of frozen (-80°C) permeabilized cells were thawed on ice and pipetted gently to fully resuspend. Aliquots were removed and permeabilized cells were counted using a Luna II, Logos Biosystems instrument. For each sample, 1 million permeabilized cells were used for nuclear run-on, with 50,000 permeabilized *Drosophila* S2 cells added to each sample for normalization. Nuclear run on assays and library preparation were performed essentially as described in Reimer et al. [57] with modifications noted: 2X nuclear run-on buffer consisted of (10 mM Tris (pH 8), 10 mM MgCl2, 1 mM DTT, 300mM KCl, 40uM/ea biotin-11-NTPs (Perkin Elmer), 0.8U/μl SuperaseIN (Thermo), 1% sarkosyl). Run-on reactions were performed at 37°C. Adenylated 3’ adapter was prepared using the 5’ DNA adenylation kit (NEB) and ligated using T4 RNA ligase 2, truncated KQ (NEB, per manufacturers instructions with 15% PEG-8000 final) and incubated at 16°C overnight. 180μl of betaine blocking buffer (1.42g of betaine brought to 10ml with binding buffer supplemented to 0.6 uM blocking oligo (TCCGACGATCCCACGTTCCCGTGG/3InvdT/)) was mixed with ligations and incubated 5 min at 65°C and 2 min on ice prior to addition of streptavidin beads. After T4 polynucleotide kinase (NEB) treatment, beads were washed once each with high salt, low salt, and blocking oligo wash (0.25X T4 RNA ligase buffer (NEB), 0.3uM blocking oligo) solutions and resuspended in 5’ adapter mix (10 pmol 5’ adapter, 30 pmol blocking oligo, water). 5’ adapter ligation was per Reimer but with 15% PEG-8000 final. Eluted cDNA was amplified 5-cycles (NEBNextμltra II Q5 master mix (NEB) with Illumina TruSeq PCR primers RP-1 and RPI-X) following the manufacturer’s suggested cycling protocol for library construction. A portion of preCR was serially diluted and for test amplification to determine optimal amplification of final libraries. Pooled libraries were sequenced using the Illumina NovaSeq platform.

#### PRO-seq data analysis

All custom scripts described herein are available on the AdelmanLab Github (https://github.com/AdelmanLab/NIH_scripts). Using a custom script (trim_and_filter_PE.pl), FASTQ read pairs were trimmed to 41bp per mate, and read pairs with a minimum average base quality score of 20 retained. Read pairs were further trimmed using cutadapt 1.14 to remove adapter sequences and low-quality 3’ bases (—match-read-wildcards -m 20 -q 10). R1 reads, corresponding to RNA 3’ ends, were then aligned to the spiked in Drosophila genome index (dm3) using Bowtie 1.2.2 (-v 2 -p 6—best—un), with those reads not mapping to the spike genome serving as input to the primary genome alignment step (using Bowtie 1.2.2 options -v 2—best). Reads mapping to the hg38 reference genome were then sorted, via samtools 1.3.1 (-n), and subsequently converted to bedGraph format using a custom script (bowtie2stdBedGraph.pl). Because R1 in PRO-seq reveals the position of the RNA 3’ end, the “+” and “-”strands were swapped to generate bedGraphs representing 3’ end position at single nucleotide resolution.

For a table of statistics, including raw read counts, mappable read counts to the spike in and reference genomes, refer to S7 Table. Pairwise correlation (Spearman’s rho) of counts in windows ±2kb around filtered TSS annotation noted in S8 Table.

For promoter reads, annotated transcription start sites were obtained from Ensembl v99 for hg38. After removing transcripts with {immunoglobulin, Mt_tRNA, Mt_rRNA} biotypes, PRO-seq signal in each sample was calculated in the window from the annotated TSS to +150 nt downstream, using a custom script, make_heatmap.pl.

Given good agreement between replicates (Spearman’s rho ≥0.95) and similar return of spike-in reads, bedGraphs were merged within conditions, and depth-normalized, to generate bigWig files binned at 10bp.

To determine differentially expressed genes in PRO-seq analyses, the 5’ ends from all PRO-seq reads were used to identify active transcription start sites using a custom script, proTSScall available on the NascentTranscriptionCore GitHub (https://github.com/NascentTranscriptionCore/proTSScall). Briefly, PRO-seq 3’ read bedGraphs for “+” and “-”strands were separately combined across samples and the composite read counts were assigned to TSS-proximal windows (TSS to +150nt) using the same filtered TSS annotation described above. TSSs with ≤9 counts in this window are deemed ‘inactive’ and the remaining TSSs, deemed ‘active’, are collapsed to yield 1 dominant TSS per gene, defined as the one with the highest TSS-proximal read count—if the highest read count is shared amongst multiple transcripts, the TSS furthest upstream, in a strand-aware fashion, is called dominant. Dominant TSSs sharing the same start position are deduplicated as follows: (1) if start positions are equal, the TSS with the longest associated annotated transcript is called dominant, (2) if start positions and transcript lengths are both equal, the TSS associated with the lowest Ensembl gene ID (numerical portion) is dominant.

To compare DEGs in our datasets to that of Rao et al. 2017 [15], we compared their list of differentially expressed genes (n = 4195) to the differentially expressed genes in our knockdowns of NIPBL (n = 1876) and WAPL (n = 1931). To evaluate whether the overlaps were larger than expected, we used one-tailed Fisher’s exact tests, using a background number of 20,000 total genes that could appear on either list to fill in the non-overlap cells of the contingency tables. Of the differentially expressed genes in the WAPL knockdown, we found 405 (21 percent) also appeared on the Rao et al. [15] list (p = 0.51). However, for the NIPBL knockdown, we found 578 genes (31 percent) also appeared on the Rao et al. list, a significant overlap (p = 3.93 x 10^-26).

#### Principle component analysis

PRO-seq 3’ reads were summed across the 2kb downstream of each TSS and genes with non-zero sums in at least one sample were retained for PCA analysis. The PCA was generated with the plotPCA function within DESeq2 using the rlog-transformed sums.

#### dREG enhancer peak calling

Enhancer peaks were called using the dREG pipeline [58] on merged PRO-seq bigwigs using the default parameters. Peaks were filtered by p-value of 0.02 or less and dREG score of 0.55 or more. Resulting peaks list was manually curated into standard bed format. Centers called outside of the dREG peak area were manually moved to the closest end of the dREG peak. dREG scores were multiplied by 1000 and converted to integers to conform to standard BED file format. Peaks assigned an “NA” p-value from DESeq2 were removed (v1.30.1) [59]. Promoter proximal dREG peaks within 1kb of an annotated TSS (Ensembl v99) were filtered using the UCSC Table Browser [60]. All other peaks were annotated as “distal”. Intragenic peaks were defined as distal dREG peaks that overlapped an annotated gene body. All others were flagged as intergenic.

#### Differential expression analysis

Differential expression analysis was performed in R v3.6.1 with DESeq2 v1.30.1 [59]. Read counts were obtained over whole genes from TSS to TES as defined by proTSScall, distal dREG peaks, TSS proximal regions (dominant TSS to TSS+150bp), and gene bodies (dominant TSS+250 to TSS+2250bp) using the featureCounts function from Rsubread v1.34.7 [61]. Defaults were used with the following exceptions: minMQS = 10; countChimericFragments = FALSE; isPairedEnd = FALSE; strandSpecific = 2 (or strandSpecific = 0 for distal dREG peaks); nthreads = 8. DESeq2 was run with defaults using the nbinomWaldTest function. The size factors obtained from whole gene bodies were applied to all other groups. Log fold change shrinkage was performed using the ‘apeglm’ algorithm [62]. Significant differentially expressed genes were filtered for a minimum adjusted p-value of 0.01 or less, removing NA values.

### ChIP-Seq

HCT116 cells were cross-linked in culture medium by addition of methanol-free formaldehyde (ThermoFisher, final 1% v/v) and incubated at room temperature for 10 minutes with gentle nutation. Crosslinking was quenched by addition of glycine (final 125 mM) and incubated at room temperature for 5 minutes with gentle nutation. Media was aspirated and replaced with cold DPBS. Cells were scraped and transferred to conical tubes, then pelleted by centrifugation (1500 rpm, 3 minutes, room temperature). Pellets were flash frozen on liquid nitrogen and stored at -80°C. For ChIP, 30 μl protein G magnetic beads (per sample; ThermoFisher) were washed three times in blocking solution (0.5% BSA in DPBS). Beads were then resuspended in 250 μl blocking solution and 2 μg antibody (RAD21, Abcam ab992; CTCF, Cell Signaling Technology 3418) was added. Beads and antibody were rotated at 4°C for at least six hours. Nuclei were isolated from frozen cell pellets as follows: pellet was resuspended in 10 mL cold lysis buffer 1 (50mM HEPES-KOH pH7.5, 140mM NaCl, 1mM EDTA, 10% Glycerol, 0.5% NP-40, 0.25% Triton X-100, and protease inhibitors) and rotated at 4°C for 10 minutes, followed by centrifugation (1500 rpm, 3 minutes, 4°C). Supernatant was aspirated and the pellet was resuspended in 10 mL cold lysis buffer 2 (10mM Tris-HCl pH 8.0, 200mM NaCl, 1mM EDTA, 0.5mM EGTA, and protease inhibitors) and rotated at room temperature for 10 minutes, followed by centrifugation (1500 rpm, 3 minutes, 4°C). Supernatant was discarded and nuclei were resuspended in 1 mL cold lysis buffer 3 (10mM Tris-HCl, pH 8.0, 100mM NaCl, 1mM EDTA, 0.5mM EGTA, 0.1% Na-Deoxycholate, and protease inhibitors) and transferred to pre-chilled 1mL Covaris AFA tubes (Covaris). Nuclei were sonicated using a Covaris S220 sonicator (high cell chromatin shearing for 15 minutes; Covaris). Sonicated chromatin was transferred to 1.5mL microcentrifuge tubes and Triton-X 100 was added (1% final v/v) followed by centrifugation (top speed, 10 minutes, 4°C). Supernatant was transferred to a new tube. Antibody-conjugated protein G beads were washed three times in blocking solution, resuspended in 50 μl blocking buffer, and added to 500 μg sonicated chromatin. Chromatin was rotated overnight at 4°C. 50 μg lysate was reserved in a separate tube at -20°C for input. On day 2, beads were washed five times in 1 mL RIPA buffer (50mM HEPES-KOH pH 7.5, 500mM LiCl, 1mM EDTA, 1% NP-40, 0.7% Na-Deoxycholate). Beads were then washed in 1 mL final wash buffer (1xTE, 50mM NaCl) for 2 minutes. Beads were finally resuspended in 210 μl elution buffer (50mM Tris-HCl pH 8.0, 10mM EDTA, 1% SDS), and chromatin was eluted from beads by agitation at 65°C for 30 minutes. 200 μl eluate was transferred to a new tube, and all samples (ChIP and input) were reverse cross-linked overnight at 65°C with agitation for between 12 and 18 hours. 200 μl 1xTE was added to all samples, and samples were treated with RNase A (final 0.2mg/mL RNase; 37°C for 2 hours) and proteinase K (final 0.2mg/mL Proteinase K; 55C for 2 hours). DNA was purified using phenol:chloroform extraction and resuspension in 10mM Tris-HCl pH 8.0.

ChIP and input DNA were quantified by Qubit (ThermoFisher) before library preparation using the NEBNext Ultra II DNA library prep kit (NEB). Samples were indexed for either single or dual-index sequencing. Library quality was assessed on Bioanalyzer (Agilent) and quantified by qPCR (Kapa Biosystems). Libraries were pooled, re-quantified, and sequenced on the Illumina NextSeq 500 platform (Illumina, single-end 75bp) or the Illumina NovaSeq 6000 platform (Illumina, paired-end 100bp).

### ChIP-seq analysis

Sequencing quality was examined using FASTQC to ensure that the library GC% and duplication rate were within expected range (v0.11.5). Reads were aligned against the hg38 reference genome using Bowtie2 (v2.2.5) allowing 1 mismatch in seed alignment ("-N 1") and soft clipping ("—local"). For single-end libraries, the primary alignment of each read with a MAPQ score higher than 10 was retained using SAMtools (v0.1.19). For paired-end reads, only properly paired primary alignment were retained and the maximum fragment size was set to be 2kb. Alignments were then filtered for PCR duplicates using SAMtools. Alignments that mapped to mitochondria, random contigs and ENCODE blacklisted regions were also removed for downstream analysis using BEDtools (v2.27.1). Visualization tracks were generated using BEDtools, in which process, samples were normalized to 1M reads per library and corresponding input controls were subtracted from IP. Peaks were called for IP libraries against their corresponding input controls using MACS2 (v2.1.1), with default parameters and a 0.01 q-value cutoff. Finally, a non-overlapping union set of peaks was created by merging peaks in all samples using BEDtools such that all peaks that overlap by at least 1bp were merged. The union peaks were annotated to nearest genes using HOMER tools. ChIP-seq signal was quantified in each sample over each of the union peaks using Bwtools. From the union set of peaks, a reference set of peaks for RAD21 and CTCF in each condition was generated from biological replicates by compiling peaks that were detected in at least two biological replicates for a given condition.

### Statistical analysis

The numbers of samples (n), p values, and specific statistical tests performed for each experiment are noted in the figure legends. Biological replicates involved an independent isolation of cells including any relevant treatment. HiDRO replicates represent separate wells of a 384-well plate. Statistical analyses were performed using Prism 9 software by GraphPad (v9.2.0).

## Supporting information

S1 Fig Additional information related to Fig 1. (A) Fluorescent western blot to NIPBL (top of the two bands) and WAPL in the whole cell lysate from RNAi control, NIPBL, or WAPL depleted HCT116 cells. (B) Mean fold change (%) in expression by qPCR for NIPBL and WAPL in each respective knockdown. Each symbol represents a biological replicate, error bars represent standard deviation. (C) Mitotic index measured by percentage of cells that stained positive for phospho-Histone H3 (PH3) by IF in RNAi control, NIPBL, or WAPL depleted HCT116 cells and HCT116-RAD21-AID cells -/+ auxin for 6 or 24 hours. Each bar represents the mean of 3 biological replicates, error bars represent standard deviation. Unpaired t test, *** p < 0.001, ** p = 0.004, ns = not significant (p = 0.23 for Control vs. NIPBL; p = 0.44 for Control vs. WAPL). (D) Average percentage of mitotic cells in each stage of mitosis in RNAi control, NIPBL, or WAPL depleted HCT116 cells and HCT116-RAD21-AID cells -/+ auxin for 6 or 24 hours. Pro. = prometaphase, Meta. = metaphase, Ana./Telo. = Anaphase or Telophase. Each bar represents the average of 3 biological replicates, error bars represent standard deviation. (E) HRP western blot to RAD21 in chromatin-bound subcellular protein fractionations of HCT116-RAD21-AID cells -/+ auxin for 6 hours. All bands from the same blot. (F) Mean fold change (%) of RAD21 bound to chromatin in HCT116-RAD21-AID cells -/+ auxin. Each symbol represents a biological replicate, error bars represent standard deviation. (G) Representative FISH images for three domains at chr2:217-222Mb in HCT116-RAD21-AID cells -/+ auxin. Dashed line represents nuclear edge, scale bar, 5μm (above) or 1μm (below). (H) Cumulative frequency distribution of overlap between the neighboring domains D1 and D2 on chr2 in HCT116-RAD21-AID cells before (n = 1,874 chromosomes) and after auxin treatment (n = 2,128 chromosomes). Two-tailed Mann-Whitney test, *** p < 0.001. (I) Cumulative frequency distribution of overlap between the neighboring domains D2 and D3 on chr2 in HCT116-RAD21-AID cells before (n = 1,898 chromosomes) and after auxin treatment (n = 2,190 chromosomes). Two-tailed Mann-Whitney test, *** p < 0.001. (J) Chromosome schematic representing the relative locations of the HiDRO Oligopaint FISH probes. (K) Oligopaint design for three neighboring domains at chr2:217-222Mb. (L) Representative FISH images for three domains at chr2:217-222Mb in HCT116-RAD21-AID cells -/+ auxin. Dashed line represents nuclear edge, scale bar, 5μm (above) or 1μm (below). (M) Change in contact frequency across 18 domain pairs in NIPBL, or WAPL depleted HCT116 cells and auxin treated HCT116-RAD21-AID cells. Each bar represents the median of ≥ 4 biological replicates. D indicates domain boundary, S indicates sub-domain boundary.
(TIF)

S2 Fig Additional information related to Fig 2. (A) Top 5 GO Biological Processes scored by adjusted p-value for NIPBL DEGs and their significance. (B) Top 5 GO Biological Processes scored by adjusted p-value for WAPL DEGs and their significance. (C) Top 5 GO Biological Processes scored by adjusted p-value for RAD21 DEGs and their significance. (D) The log_2_(fold change) of shared DEGs across NIPBL and WAPL knockdown conditions. (E) Percentage of up, down, NIPBL, WAPL, or nonDEGs with a TSS within 5kb of a RAD21 ChIP-Seq peak co-occupied by CTCF. Fisher’s exact test, **** p < 0.0001, *** p = 0.0002.
(TIF)

S3 Fig Additional information related to Fig 3. (A) The number of expected versus observed TADs with binned coordination scores. 50% coordination represents random misexpression of NIPBL DEGs and 100% coordination represents all NIPBL DEGs in the TAD being up or down regulated. The dot represents the observed data, compared to the expected data in the null distribution (violin plot) generated by shuffling the fold change values amongst the DEGs 1,000 times. (B) The number of expected versus observed TADs with binned coordination scores. The dot represents the observed data, compared to the expected data in the null distribution (violin plot) generated by shuffling the fold change values amongst the DEGs 1,000 times. (C) The log_2_(fold change) of dREG peaks after NIPBL knockdown versus their significance. Significantly changed dREG peaks are in green (n = 85) and non-significantly changed dREG peaks (adjusted p-value > 0.01) are in grey (n = 19,234). (D) The log_2_(fold change) of dREG peaks after WAPL knockdown versus their significance. Significantly changed dREG peaks are in green (n = 226) and non-significantly changed dREG peaks (adjusted p-value > 0.01) are in grey (n = 21,732).
(TIF)

S4 Fig Additional information related to Fig 4. (A) Cell growth measured in 24-hour increments following RNAi to NIPBL and WAPL or a non-targeting sequence as the control. Each bar represents the mean of 3 biological replicates and error bars represent the standard deviation. (B) Mitotic index measured by percentage of cells that stained positive for phospho-Histone H3 (PH3) by IF in RNAi control or NIPBL and WAPL depleted HCT116 cells. Each bar represents the mean of 3 biological replicates, error bars represent standard deviation. Unpaired t test, ns = not significant (p = 0.94). (C) Representative immunofluorescence images of mitotic cells stained for α-tubulin (cyan) and phospho-Histone H3 (PH3; red) in RNAi control or NIPBL and WAPL depleted HCT116 cells. Scale bar, 5μm. (D) Average percentage of mitotic cells with abnormal mitosis in RNAi control or NIPBL and WAPL depleted HCT116 cells. Each symbol represents a biological replicate, error bars represent standard deviation. (E) Cumulative frequency distribution of overlap between the neighboring domains D1 and D2 on chr2 in RNAi control (n = 1,954 chromosomes), NIPBL (n = 1,584 chromosomes), WAPL (n = 1,677 chromosomes), or dKD (n = 1,711 chromosomes) depleted HCT116 cells. Two-tailed Mann-Whitney test, **** p < 0.0001, ** p = 0.0012. Biological replicate of data in Fig 4G. (F) Cumulative frequency distribution of overlap between the neighboring domains D2 and D3 on chr2 in RNAi control (n = 1,956 chromosomes), NIPBL (n = 1,671 chromosomes), WAPL (n = 1,666 chromosomes), or dKD (n = 1,728 chromosomes) depleted HCT116 cells. Two-tailed Mann-Whitney test, **** p < 0.0001, ns = not significant (p = 0.18). Biological replicate of data in Fig 4I. (G) Change in contact frequency across 18 domain pairs in HCT116 cells depleted for NIPBL, WAPL, or both. Each bar represents the median of ≥ 4 biological replicates. D indicates domain boundary; S indicates sub-domain boundary.
(TIF)

S5 Fig Additional information related to Fig 5. (A) The log_2_(fold change) of genes after CTCF knockdown versus their significance. DEGs are in blue (2,002 up, 1,887 down) and non-significantly changed genes (adjusted p-value > 0.01) are in grey. (B) Venn diagram of the NIPBL, WAPL, and CTCF DEGs. (C) Number of NIPBL DEGs fully, partially, or not rescued in the NIPBL/CTCF double knockdown condition.
(TIF)

S1 Table Biological processes associated with NIPBL knockdown. Top 10 GO Biological Processes for NIPBL DEGs sorted by adjusted p-value.
(DOCX)

S2 Table Biological processes associated with RAD21 knockdown. Top 10 GO Biological Processes for RAD21 DEGs sorted by adjusted p-value.
(DOCX)

S3 Table Biological processes associated with WAPL knockdown. Top 10 GO Biological Processes for WAPL DEGs sorted by adjusted p-value.
(DOCX)

S4 Table NIPBL knockdown-associated biological processes rescued by co-depletion with WAPL. Top 10 GO Biological Processes for NIPBL DEGs rescued in the double knockdown condition sorted by adjusted p-value.
(DOCX)

S5 Table WAPL knockdown-associated biological processes rescued by co-depletion with NIPBL. Top 10 GO Biological Processes for WAPL DEGs rescued in the double knockdown condition sorted by adjusted p-value.
(DOCX)

S6 Table Oligopaint design. Oligopaint design coordinates (hg19) and probe densities.
(DOCX)

S7 Table PRO-seq statistics. PRO-seq statistics, including raw read counts, mappable read counts to the spike in and reference genomes.
(DOCX)

S8 Table Pairwise correlation of PRO-seq counts. Pairwise correlation (Spearman’s rho) of PRO-seq counts in windows ±2kb around filtered TSS annotation. N represents NIPBL knockdown, W represents WAPL knockdown, C represents CTCF knockdown, NW represents NIPBL and WAPL double knockdown, and NC represents NIPBL and CTCF double knockdown.
(DOCX)
