# Supplementary figures and images for: Co-depletion of NIPBL and WAPL balance cohesin activity to correct gene misexpression
Source: PLoS Genet. 2022 Nov 30;18(11):e1010528. doi: 10.1371/journal.pgen.1010528 (PMC9744307; doi:10.1371/journal.pgen.1010528)

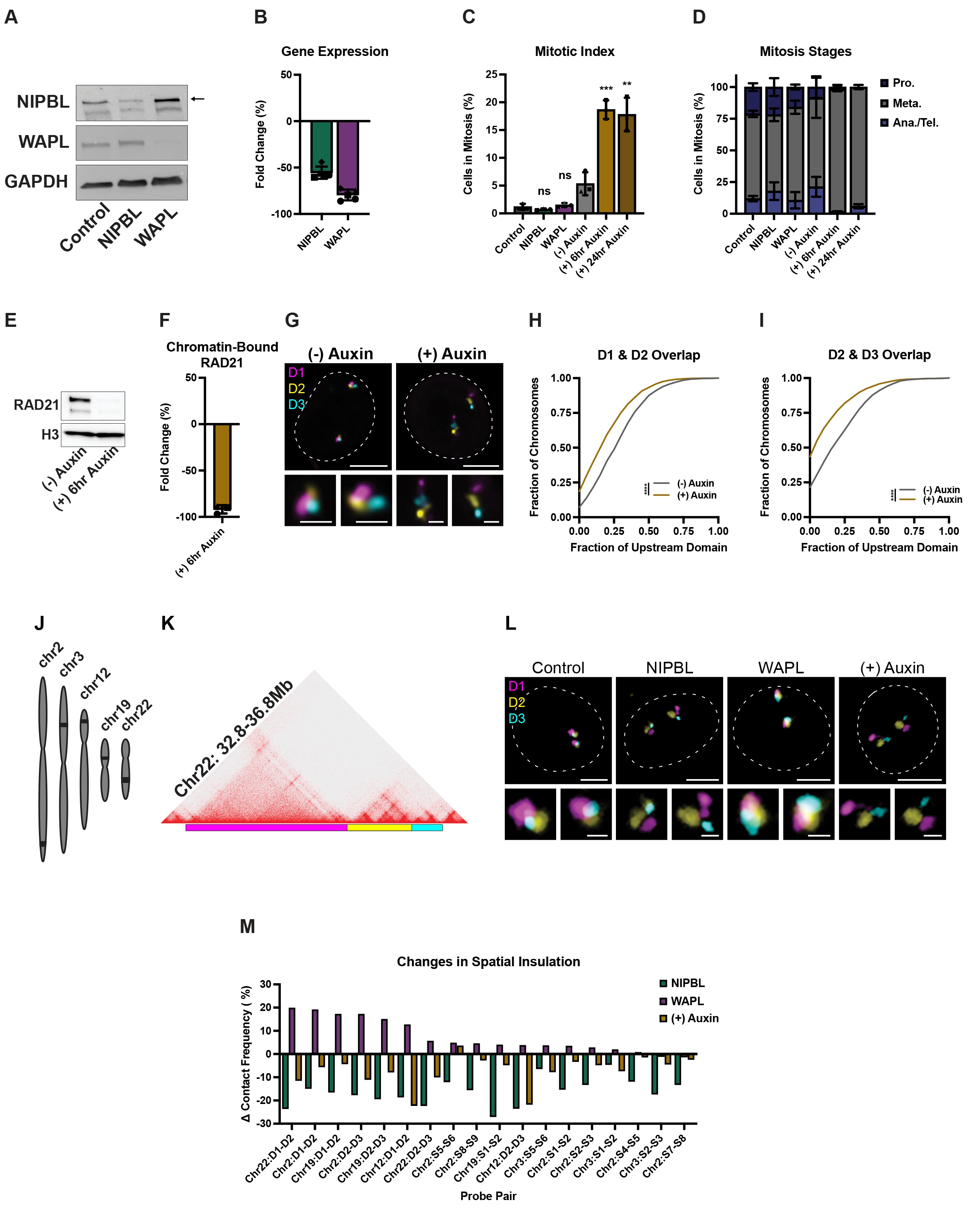

Supplement: S1 Fig — (A) Fluorescent western blot to NIPBL (top of the two bands) and WAPL in the whole cell lysate from RNAi control, NIPBL, or WAPL depleted HCT116 cells. (B) Mean fold change (%) in expression by qPCR for NIPBL and WAPL in each respective knockdown. Each symbol represents a biological replicate, error bars represent standard deviation. (C) Mitotic index measured by percentage of cells that stained positive for phospho-Histone H3 (PH3) by IF in RNAi control, NIPBL, or WAPL depleted HCT116 cells and HCT116-RAD21-AID cells -/+ auxin for 6 or 24 hours. Each bar represents the mean of 3 biological replicates, error bars represent standard deviation. Unpaired t test, *** p < 0.001, ** p = 0.004, ns = not significant (p = 0.23 for Control vs. NIPBL; p = 0.44 for Control vs. WAPL). (D) Average percentage of mitotic cells in each stage of mitosis in RNAi control, NIPBL, or WAPL depleted HCT116 cells and HCT116-RAD21-AID cells -/+ auxin for 6 or 24 hours. Pro. = prometaphase, Meta. = metaphase, Ana./Telo. = Anaphase or Telophase. Each bar represents the average of 3 biological replicates, error bars represent standard deviation. (E) HRP western blot to RAD21 in chromatin-bound subcellular protein fractionations of HCT116-RAD21-AID cells -/+ auxin for 6 hours. All bands from the same blot. (F) Mean fold change (%) of RAD21 bound to chromatin in HCT116-RAD21-AID cells -/+ auxin. Each symbol represents a biological replicate, error bars represent standard deviation. (G) Representative FISH images for three domains at chr2:217-222Mb in HCT116-RAD21-AID cells -/+ auxin. Dashed line represents nuclear edge, scale bar, 5μm (above) or 1μm (below). (H) Cumulative frequency distribution of overlap between the neighboring domains D1 and D2 on chr2 in HCT116-RAD21-AID cells before (n = 1,874 chromosomes) and after auxin treatment (n = 2,128 chromosomes). Two-tailed Mann-Whitney test, *** p < 0.001. (I) Cumulative frequency distribution of overlap between the neighboring domains D2 and D3 o [file pgen.1010528.s001.tif]

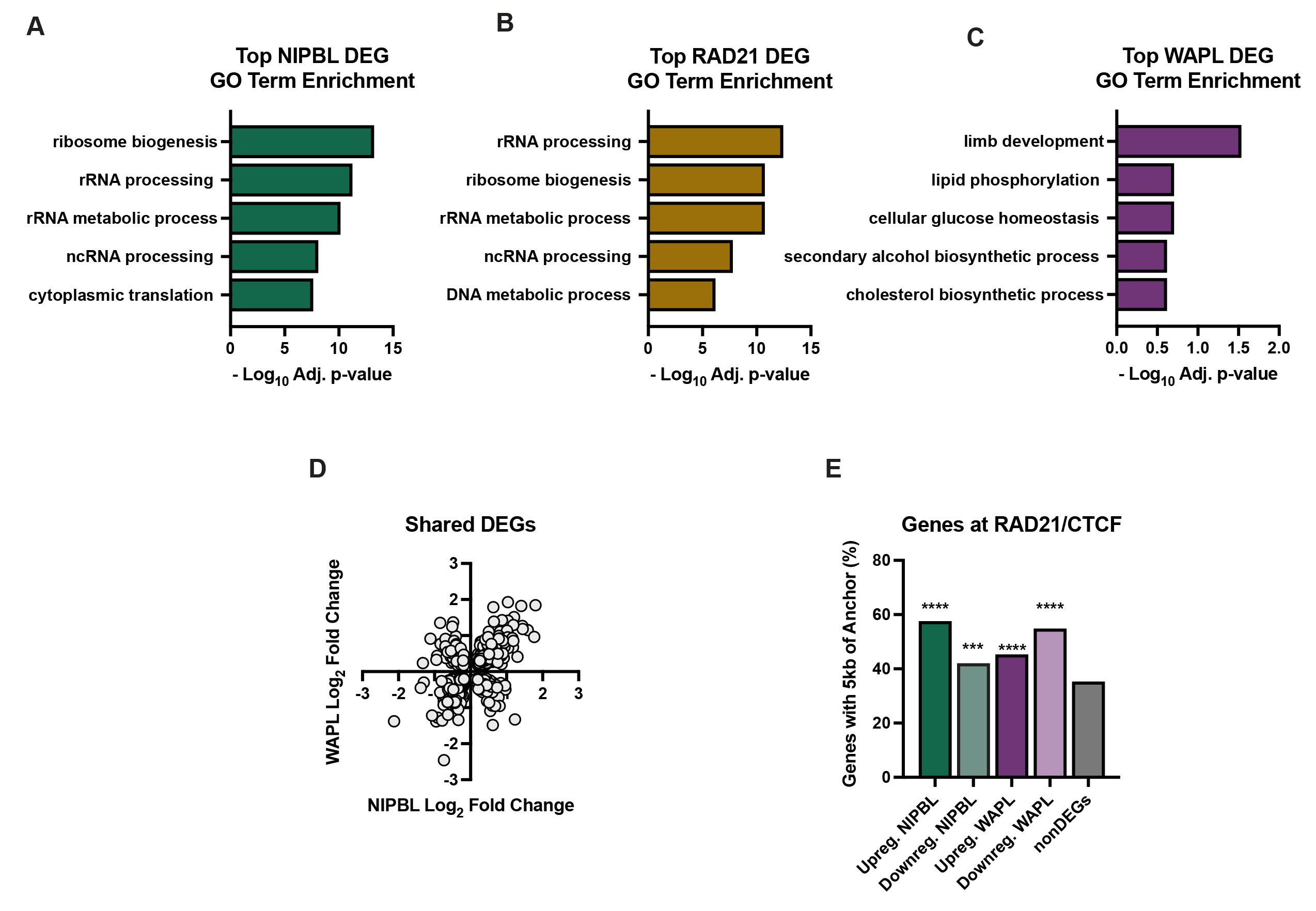

Supplement: S2 Fig — (A) Top 5 GO Biological Processes scored by adjusted p-value for NIPBL DEGs and their significance. (B) Top 5 GO Biological Processes scored by adjusted p-value for WAPL DEGs and their significance. (C) Top 5 GO Biological Processes scored by adjusted p-value for RAD21 DEGs and their significance. (D) The log2(fold change) of shared DEGs across NIPBL and WAPL knockdown conditions. (E) Percentage of up, down, NIPBL, WAPL, or nonDEGs with a TSS within 5kb of a RAD21 ChIP-Seq peak co-occupied by CTCF. Fisher’s exact test, **** p < 0.0001, *** p = 0.0002. (TIF) [file pgen.1010528.s002.tif]

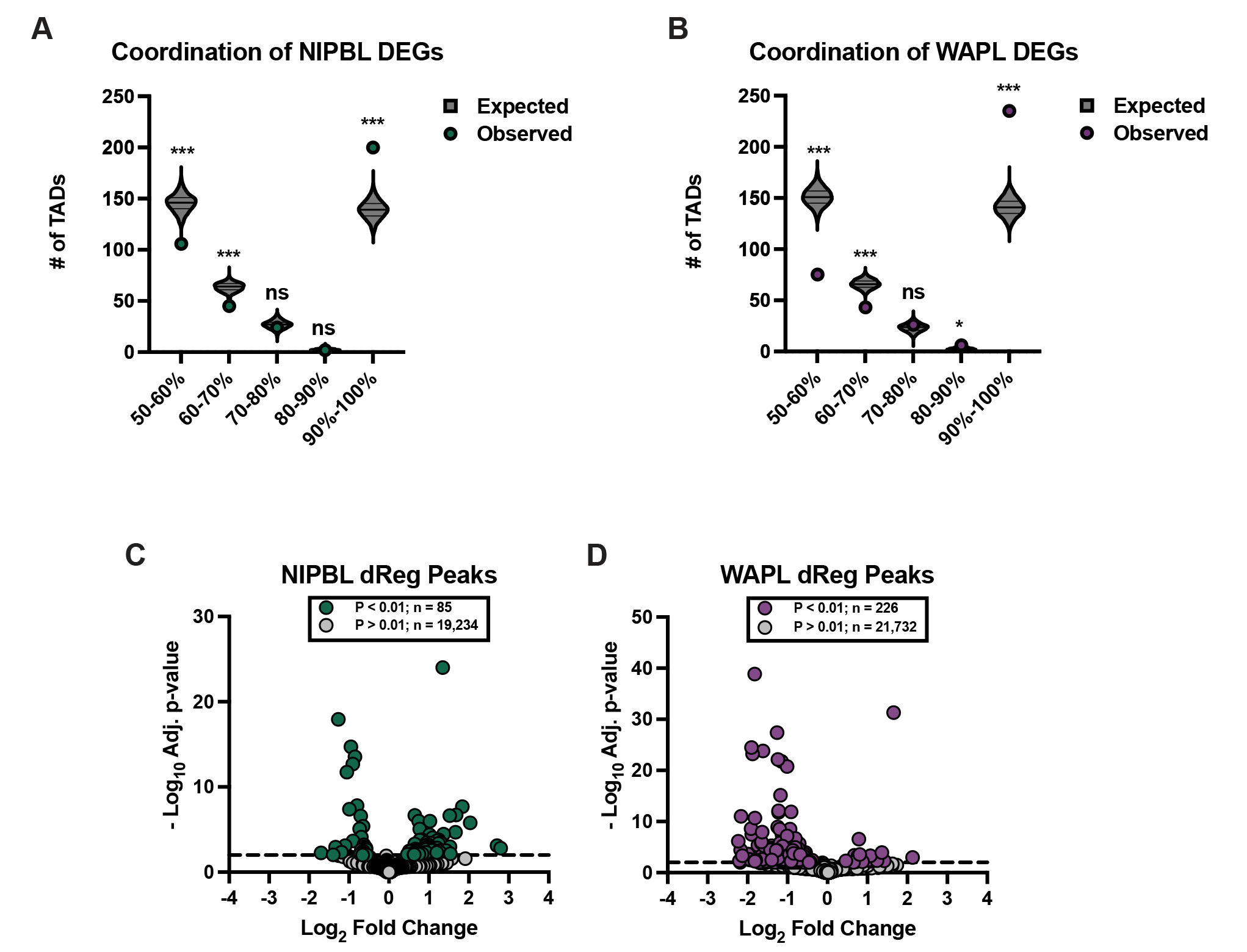

Supplement: S3 Fig — (A) The number of expected versus observed TADs with binned coordination scores. 50% coordination represents random misexpression of NIPBL DEGs and 100% coordination represents all NIPBL DEGs in the TAD being up or down regulated. The dot represents the observed data, compared to the expected data in the null distribution (violin plot) generated by shuffling the fold change values amongst the DEGs 1,000 times. (B) The number of expected versus observed TADs with binned coordination scores. The dot represents the observed data, compared to the expected data in the null distribution (violin plot) generated by shuffling the fold change values amongst the DEGs 1,000 times. (C) The log2(fold change) of dREG peaks after NIPBL knockdown versus their significance. Significantly changed dREG peaks are in green (n = 85) and non-significantly changed dREG peaks (adjusted p-value > 0.01) are in grey (n = 19,234). (D) The log2(fold change) of dREG peaks after WAPL knockdown versus their significance. Significantly changed dREG peaks are in green (n = 226) and non-significantly changed dREG peaks (adjusted p-value > 0.01) are in grey (n = 21,732). (TIF) [file pgen.1010528.s003.tif]

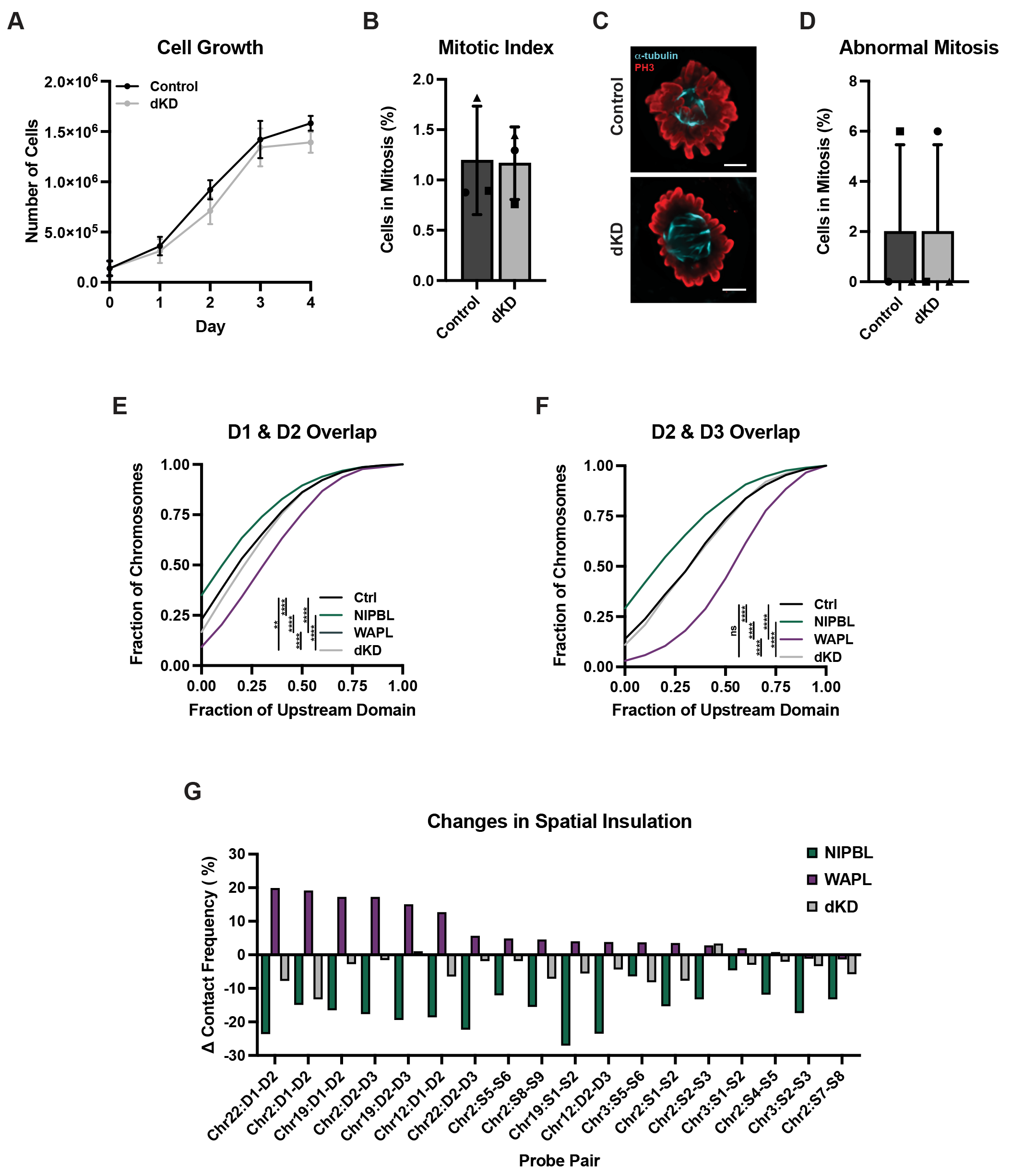

Supplement: S4 Fig — (A) Cell growth measured in 24-hour increments following RNAi to NIPBL and WAPL or a non-targeting sequence as the control. Each bar represents the mean of 3 biological replicates and error bars represent the standard deviation. (B) Mitotic index measured by percentage of cells that stained positive for phospho-Histone H3 (PH3) by IF in RNAi control or NIPBL and WAPL depleted HCT116 cells. Each bar represents the mean of 3 biological replicates, error bars represent standard deviation. Unpaired t test, ns = not significant (p = 0.94). (C) Representative immunofluorescence images of mitotic cells stained for α-tubulin (cyan) and phospho-Histone H3 (PH3; red) in RNAi control or NIPBL and WAPL depleted HCT116 cells. Scale bar, 5μm. (D) Average percentage of mitotic cells with abnormal mitosis in RNAi control or NIPBL and WAPL depleted HCT116 cells. Each symbol represents a biological replicate, error bars represent standard deviation. (E) Cumulative frequency distribution of overlap between the neighboring domains D1 and D2 on chr2 in RNAi control (n = 1,954 chromosomes), NIPBL (n = 1,584 chromosomes), WAPL (n = 1,677 chromosomes), or dKD (n = 1,711 chromosomes) depleted HCT116 cells. Two-tailed Mann-Whitney test, **** p < 0.0001, ** p = 0.0012. Biological replicate of data in Fig 4G. (F) Cumulative frequency distribution of overlap between the neighboring domains D2 and D3 on chr2 in RNAi control (n = 1,956 chromosomes), NIPBL (n = 1,671 chromosomes), WAPL (n = 1,666 chromosomes), or dKD (n = 1,728 chromosomes) depleted HCT116 cells. Two-tailed Mann-Whitney test, **** p < 0.0001, ns = not significant (p = 0.18). Biological replicate of data in Fig 4I. (G) Change in contact frequency across 18 domain pairs in HCT116 cells depleted for NIPBL, WAPL, or both. Each bar represents the median of ≥ 4 biological replicates. D indicates domain boundary; S indicates sub-domain boundary. (TIF) [file pgen.1010528.s004.tif]

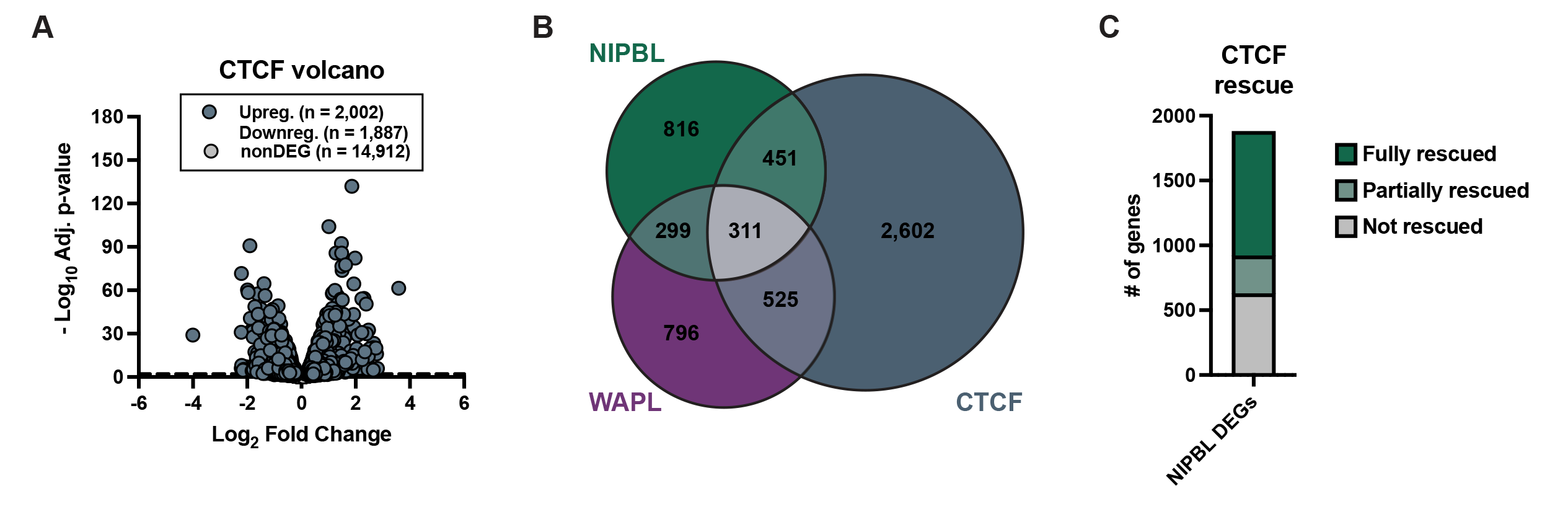

Supplement: S5 Fig — (A) The log2(fold change) of genes after CTCF knockdown versus their significance. DEGs are in blue (2,002 up, 1,887 down) and non-significantly changed genes (adjusted p-value > 0.01) are in grey. (B) Venn diagram of the NIPBL, WAPL, and CTCF DEGs. (C) Number of NIPBL DEGs fully, partially, or not rescued in the NIPBL/CTCF double knockdown condition. (TIF) [file pgen.1010528.s005.tif]
