# Supplementary material for: Co-depletion of NIPBL and WAPL balance cohesin activity to correct gene misexpression
Source: PLoS Genet. 2022 Nov 30;18(11):e1010528. doi: 10.1371/journal.pgen.1010528 (PMC9744307; doi:10.1371/journal.pgen.1010528)
Supplement: S1 Table — Top 10 GO Biological Processes for NIPBL DEGs sorted by adjusted p-value. (DOCX) [file pgen.1010528.s006.docx]

**S1 Table. Biological processes associated with NIPBL knockdown.**

Top 10 GO Biological Processes for NIPBL DEGs sorted by adjusted p-value.

| **Term** | **P-value** | **Adjusted P-value** | **Odds Ratio** | **Combined Score** |
| --- | --- | --- | --- | --- |
| ribosome biogenesis (GO:0042254) | 1.45E-17 | 6.31E-14 | 4.50067544 | 174.497863 |
| rRNA processing (GO:0006364) | 2.85E-15 | 6.18E-12 | 4.35927906 | 146.003978 |
| rRNA metabolic process (GO:0016072) | 5.49E-14 | 7.95E-11 | 4.27223524 | 130.446561 |
| ncRNA processing (GO:0034470) | 8.12E-12 | 8.82E-09 | 3.43715363 | 87.7742124 |
| cytoplasmic translation (GO:0002181) | 2.94E-11 | 2.55E-08 | 5.13558132 | 124.540786 |
| SRP-dependent cotranslational protein targeting to membrane (GO:0006614) | 2.96E-10 | 2.14E-07 | 4.88982133 | 107.288749 |
| cellular macromolecule biosynthetic process (GO:0034645) | 7.71E-10 | 4.78E-07 | 2.57500199 | 54.0332499 |
| cotranslational protein targeting to membrane (GO:0006613) | 9.78E-10 | 5.31E-07 | 4.58319234 | 95.0825749 |
| nuclear-transcribed mRNA catabolic process, nonsense-mediated decay (GO:0000184) | 1.90E-09 | 9.16E-07 | 4.0361917 | 81.0588175 |
| protein targeting to ER (GO:0045047) | 2.60E-09 | 1.13E-06 | 4.21016161 | 83.2329204 |
