# Supplementary material for: Co-depletion of NIPBL and WAPL balance cohesin activity to correct gene misexpression
Source: PLoS Genet. 2022 Nov 30;18(11):e1010528. doi: 10.1371/journal.pgen.1010528 (PMC9744307; doi:10.1371/journal.pgen.1010528)
Supplement: S2 Table — Top 10 GO Biological Processes for RAD21 DEGs sorted by adjusted p-value. (DOCX) [file pgen.1010528.s007.docx]

**S2 Table. Biological processes associated with RAD21 knockdown.**

Top 10 GO Biological Processes for RAD21 DEGs sorted by adjusted p-value.

| **Term** | **P-value** | **Adjusted P-value** | **Odds Ratio** | **Combined Score** |
| --- | --- | --- | --- | --- |
| rRNA processing (GO:0006364) | 9.89E-17 | 4.06E-13 | 4.90866134 | 180.895077 |
| ribosome biogenesis (GO:0042254) | 1.09E-14 | 2.04E-11 | 4.24279385 | 136.412063 |
| rRNA metabolic process (GO:0016072) | 1.49E-14 | 2.04E-11 | 4.64303386 | 147.817559 |
| ncRNA processing (GO:0034470) | 1.70E-11 | 1.75E-08 | 3.56823593 | 88.4781164 |
| DNA metabolic process (GO:0006259) | 8.35E-10 | 6.86E-07 | 2.83316826 | 59.2224829 |
| DNA replication (GO:0006260) | 1.89E-08 | 1.30E-05 | 4.05924636 | 72.1858683 |
| mitotic DNA replication (GO:1902969) | 2.06E-06 | 0.00121026 | 26.81875 | 351.103019 |
| pre-replicative complex assembly involved in nuclear cell cycle DNA replication (GO:0006267) | 6.48E-06 | 0.00332766 | 34.4615865 | 411.703503 |
| double-strand break repair via break-induced replication (GO:0000727) | 1.18E-05 | 0.00537649 | 16.0895 | 182.602746 |
| regulation of cyclin-dependent protein kinase activity (GO:1904029) | 1.51E-05 | 0.00620547 | 4.43419018 | 49.2214222 |
