# Supplementary material for: Co-depletion of NIPBL and WAPL balance cohesin activity to correct gene misexpression
Source: PLoS Genet. 2022 Nov 30;18(11):e1010528. doi: 10.1371/journal.pgen.1010528 (PMC9744307; doi:10.1371/journal.pgen.1010528)
Supplement: S3 Table — Top 10 GO Biological Processes for WAPL DEGs sorted by adjusted p-value. (DOCX) [file pgen.1010528.s008.docx]

**S3 Table. Biological processes associated with WAPL knockdown.**

Top 10 GO Biological Processes for WAPL DEGs sorted by adjusted p-value.

| **Term** | **P-value** | **Adjusted P-value** | **Odds Ratio** | **Combined Score** |
| --- | --- | --- | --- | --- |
| limb development (GO:0060173) | 6.87E-06 | 0.02950494 | 6.64022316 | 78.9432037 |
| lipid phosphorylation (GO:0046834) | 1.20E-04 | 0.19885464 | 14.0766234 | 127.106901 |
| cellular glucose homeostasis (GO:0001678) | 1.39E-04 | 0.19885464 | 4.07554745 | 36.1990471 |
| secondary alcohol biosynthetic process (GO:1902653) | 2.31E-04 | 0.24329865 | 4.49515399 | 37.6313707 |
| cholesterol biosynthetic process (GO:0006695) | 3.08E-04 | 0.24329865 | 4.30761719 | 34.8261574 |
| positive regulation of release of cytochrome c from mitochondria (GO:0090200) | 3.48E-04 | 0.24329865 | 5.28346124 | 42.0731483 |
| extracellular structure organization (GO:0043062) | 4.13E-04 | 0.24329865 | 1.95244613 | 15.2112593 |
| external encapsulating structure organization (GO:0045229) | 4.53E-04 | 0.24329865 | 1.94149067 | 14.9484369 |
| extracellular matrix organization (GO:0030198) | 6.16E-04 | 0.29117693 | 1.75673657 | 12.9851858 |
| sterol biosynthetic process (GO:0016126) | 6.78E-04 | 0.29117693 | 3.82835648 | 27.9342874 |
