# Supplementary material for: Co-depletion of NIPBL and WAPL balance cohesin activity to correct gene misexpression
Source: PLoS Genet. 2022 Nov 30;18(11):e1010528. doi: 10.1371/journal.pgen.1010528 (PMC9744307; doi:10.1371/journal.pgen.1010528)
Supplement: S4 Table — Top 10 GO Biological Processes for NIPBL DEGs rescued in the double knockdown condition sorted by adjusted p-value. (DOCX) [file pgen.1010528.s009.docx]

**S4 Table. NIPBL knockdown-associated biological processes rescued by co-depletion with WAPL.**

Top 10 GO Biological Processes for NIPBL DEGs rescued in the double knockdown condition sorted by adjusted p-value.

| **Term** | **P-value** | **Adjusted P-value** | **Odds Ratio** | **Combined Score** |
| --- | --- | --- | --- | --- |
| ribosome biogenesis (GO:0042254) | 2.79E-20 | 1.14E-16 | 5.277099 | 237.598868 |
| rRNA processing (GO:0006364) | 2.73E-18 | 5.58E-15 | 5.23727302 | 211.804312 |
| rRNA metabolic process (GO:0016072) | 9.23E-17 | 1.26E-13 | 5.13061397 | 189.429757 |
| ncRNA processing (GO:0034470) | 1.42E-14 | 1.45E-11 | 4.12911308 | 131.650349 |
| cytoplasmic translation (GO:0002181) | 3.79E-13 | 3.10E-10 | 6.15680228 | 176.092158 |
| SRP-dependent cotranslational protein targeting to membrane (GO:0006614) | 5.22E-12 | 3.56E-09 | 5.86102236 | 152.25747 |
| cotranslational protein targeting to membrane (GO:0006613) | 1.83E-11 | 1.01E-08 | 5.49351038 | 135.820725 |
| cellular macromolecule biosynthetic process (GO:0034645) | 1.98E-11 | 1.01E-08 | 2.91338179 | 71.8045298 |
| nuclear-transcribed mRNA catabolic process, nonsense-mediated decay (GO:0000184) | 1.33E-10 | 6.02E-08 | 4.63154902 | 105.336417 |
| ribosome biogenesis (GO:0042254) | 2.79E-20 | 1.14E-16 | 5.277099 | 237.598868 |
