# Supplementary material for: Co-depletion of NIPBL and WAPL balance cohesin activity to correct gene misexpression
Source: PLoS Genet. 2022 Nov 30;18(11):e1010528. doi: 10.1371/journal.pgen.1010528 (PMC9744307; doi:10.1371/journal.pgen.1010528)
Supplement: S5 Table — Top 10 GO Biological Processes for WAPL DEGs rescued in the double knockdown condition sorted by adjusted p-value. (DOCX) [file pgen.1010528.s010.docx]

**S5 Table.** **WAPL knockdown-associated biological processes rescued by co-depletion with NIPBL.**

Top 10 GO Biological Processes for WAPL DEGs rescued in the double knockdown condition sorted by adjusted p-value.

| **Term** | **P-value** | **Adjusted P-value** | **Odds Ratio** | **Combined Score** |
| --- | --- | --- | --- | --- |
| limb development (GO:0060173) | 1.26E-05 | 0.051100654 | 6.653472222 | 75.05731346 |
| lipid phosphorylation (GO:0046834) | 5.64E-05 | 0.094816501 | 16.29525223 | 159.4117298 |
| secondary alcohol biosynthetic process (GO:1902653) | 7.02E-05 | 0.094816501 | 5.205641822 | 49.78761941 |
| cholesterol biosynthetic process (GO:0006695) | 9.46E-05 | 0.095829197 | 4.988467262 | 46.22243612 |
| positive regulation of release of cytochrome c from mitochondria (GO:0090200) | 1.27E-04 | 0.102533187 | 6.117605529 | 54.90610128 |
| sterol biosynthetic process (GO:0016126) | 2.15E-04 | 0.145431921 | 4.433465608 | 37.43287445 |
| diacylglycerol metabolic process (GO:0046339) | 3.96E-04 | 0.205118208 | 5.797266786 | 45.41387799 |
| isoprenoid biosynthetic process (GO:0008299) | 4.05E-04 | 0.205118208 | 13.57132266 | 106.0150084 |
| peroxisome proliferator activated receptor signaling pathway (GO:0035357) | 6.65E-04 | 0.254440996 | 21.70361589 | 158.7927497 |
| cellular glucose homeostasis (GO:0001678) | 6.95E-04 | 0.254440996 | 3.739713542 | 27.19483982 |
