# Supplementary material for: Co-depletion of NIPBL and WAPL balance cohesin activity to correct gene misexpression
Source: PLoS Genet. 2022 Nov 30;18(11):e1010528. doi: 10.1371/journal.pgen.1010528 (PMC9744307; doi:10.1371/journal.pgen.1010528)
Supplement: S6 Table — Oligopaint design coordinates (hg19) and probe densities. (DOCX) [file pgen.1010528.s011.docx]

**S6 Table. Oligopaint design.**

Oligopaint design coordinates (hg19) and probe densities.

| **Probe** | **Chr** | **Start** | **Stop** | **# of Probes** | **Probes /kb** |
| --- | --- | --- | --- | --- | --- |
| Chr2:D1 | chr2 | 217531474 | 218621456 | 5464 | 5.01 |
| Chr2:D2 | chr2 | 218621456 | 220602102 | 8938 | 4.51 |
| Chr2:D3 | chr2 | 220602102 | 222889819 | 9479 | 4.14 |
| Chr2:S1 | chr2 | 219271354 | 219513415 | 765 | 3.16 |
| Chr2:S2 | chr2 | 219513415 | 219717782 | 846 | 4.14 |
| Chr2:S3 | chr2 | 219717782 | 219860895 | 656 | 4.58 |
| Chr2:S4 | chr2 | 219860895 | 220025303 | 939 | 5.71 |
| Chr2:S5 | chr2 | 220025303 | 220267857 | 1088 | 4.49 |
| Chr2:S6 | chr2 | 220267857 | 220406620 | 841 | 6.06 |
| Chr2:S7 | chr2 | 221552089 | 221965637 | 1640 | 3.97 |
| Chr2:S8 | chr2 | 221965637 | 222438280 | 2300 | 4.87 |
| Chr2:S9 | chr2 | 222438280 | 222889819 | 1635 | 3.62 |
| Chr3:S1 | chr3 | 45541203 | 45706813 | 678 | 4.09 |
| Chr3:S2 | chr3 | 45706813 | 45948482 | 1037 | 4.29 |
| Chr3:S3 | chr3 | 45948482 | 46120405 | 774 | 4.50 |
| Chr3:S5 | chr3 | 46705342 | 47040191 | 1763 | 5.27 |
| Chr3:S6 | chr3 | 47040191 | 47481841 | 1679 | 3.80 |
| Chr12:D1 | chr12 | 11707040 | 12890169 | 4676 | 3.95 |
| Chr12:D2 | chr12 | 12890169 | 13408925 | 2210 | 4.26 |
| Chr12:D3 | chr12 | 13408925 | 14338981 | 4211 | 4.53 |
| Chr19:D2 | chr19 | 17966474 | 18551439 | 2054 | 3.51 |
| Chr19:D3 | chr19 | 18551439 | 19097717 | 2502 | 4.58 |
| Chr19:S1 | chr19 | 17502026 | 17733289 | 679 | 2.94 |
| Chr19:S2 | chr19 | 17733289 | 17886271 | 514 | 3.36 |
| Chr22:D1 | chr22 | 33414401 | 35627637 | 7555 | 3.41 |
| Chr22:D2 | chr22 | 35627637 | 36520199 | 3271 | 3.66 |
| Chr22:D3 | chr22 | 36520199 | 36942436 | 1603 | 3.80 |
| MCM5 | chr22 | 35796115 | 35820495 | 120 | 4.92 |
| Chr22: part D2 | chr22 | 36019409 | 36520199 | 1673 | 3.34 |
