# Supplementary material for: Co-depletion of NIPBL and WAPL balance cohesin activity to correct gene misexpression
Source: PLoS Genet. 2022 Nov 30;18(11):e1010528. doi: 10.1371/journal.pgen.1010528 (PMC9744307; doi:10.1371/journal.pgen.1010528)
Supplement: S7 Table — PRO-seq statistics, including raw read counts, mappable read counts to the spike in and reference genomes. (DOCX) [file pgen.1010528.s012.docx]

**S7 Table.** **PRO-seq statistics.**

PRO-seq statistics, including raw read counts, mappable read counts to the spike in and reference genomes.

| **Samples** | **Number Raw Reads** | **Number Spike Reads Mapped** | **Number Unique Ref Reads Mapped** |
| --- | --- | --- | --- |
| PR28_control1 | 81763555 | 15317608 | 50020778 |
| PR30_control2 | 96354537 | 17005602 | 67235834 |
| PR32_Nipbl1 | 71081725 | 14234691 | 46820905 |
| PR34_Nipbl2 | 86026983 | 15549770 | 61607767 |
| PR33_Wapl1 | 90247992 | 18205253 | 51629855 |
| PR35_Wapl2 | 75712441 | 13925115 | 51202452 |
| PR29_NipblWapl1 | 88589172 | 14890161 | 61330222 |
| PR31_NipblWapl2 | 82623691 | 16999655 | 57261439 |
| PR36_NipblCtcf1 | 87588790 | 12047409 | 52428882 |
| PR38_NipblCtcf2 | 86547017 | 13443008 | 64122493 |
| PR37_Ctcf1 | 93071126 | 18664948 | 61286428 |
| PR39_Ctcf2 | 91546835 | 15866768 | 65933384 |
