# Supplementary material for: Co-depletion of NIPBL and WAPL balance cohesin activity to correct gene misexpression
Source: PLoS Genet. 2022 Nov 30;18(11):e1010528. doi: 10.1371/journal.pgen.1010528 (PMC9744307; doi:10.1371/journal.pgen.1010528)
Supplement: S8 Table — Pairwise correlation (Spearman’s rho) of PRO-seq counts in windows ±2kb around filtered TSS annotation. N represents NIPBL knockdown, W represents WAPL knockdown, C represents CTCF knockdown, NW represents NIPBL and WAPL double knockdown, and NC represents NIPBL and CTCF double knockdown. (DOCX) [file pgen.1010528.s013.docx]

**S8 Table.** **Pairwise correlation of PRO-seq counts.**

Pairwise correlation (Spearman's rho) of PRO-seq counts in windows ±2kb around filtered TSS annotation. N represents NIPBL knockdown, W represents WAPL knockdown, C represents CTCF knockdown, NW represents NIPBL and WAPL double knockdown, and NC represents NIPBL and CTCF double knockdown.

|  | **WT1** | **NW1** | **N1** | **W1** | **NC1** | **C1** | **WT2** | **NW2** | **N2** | **W2** | **NC2** | **C2** |
| --- | --- | --- | --- | --- | --- | --- | --- | --- | --- | --- | --- | --- |
| **WT1** | NA | NA | NA | NA | NA | NA | NA | NA | NA | NA | NA | NA |
| **NW1** | 0.96 | NA | NA | NA | NA | NA | NA | NA | NA | NA | NA | NA |
| **N1** | 0.96 | 0.96 | NA | NA | NA | NA | NA | NA | NA | NA | NA | NA |
| **W1** | 0.96 | 0.96 | 0.96 | NA | NA | NA | NA | NA | NA | NA | NA | NA |
| **NC1** | 0.95 | 0.95 | 0.95 | 0.95 | NA | NA | NA | NA | NA | NA | NA | NA |
| **C1** | 0.96 | 0.96 | 0.96 | 0.95 | 0.96 | NA | NA | NA | NA | NA | NA | NA |
| **WT2** | 0.96 | 0.96 | 0.96 | 0.96 | 0.95 | 0.96 | NA | NA | NA | NA | NA | NA |
| **NW2** | 0.96 | 0.96 | 0.96 | 0.96 | 0.95 | 0.95 | 0.96 | NA | NA | NA | NA | NA |
| **N2** | 0.96 | 0.96 | 0.96 | 0.96 | 0.95 | 0.96 | 0.96 | 0.96 | NA | NA | NA | NA |
| **W2** | 0.96 | 0.96 | 0.96 | 0.96 | 0.95 | 0.95 | 0.96 | 0.96 | 0.96 | NA | NA | NA |
| **NC2** | 0.95 | 0.96 | 0.95 | 0.95 | 0.96 | 0.96 | 0.96 | 0.95 | 0.96 | 0.95 | NA | NA |
| **C2** | 0.96 | 0.96 | 0.95 | 0.96 | 0.96 | 0.96 | 0.96 | 0.96 | 0.96 | 0.96 | 0.96 | NA |
